# A phylogenomic and comparative genomic analysis of *Commensalibacter*, a versatile insect symbiont

**DOI:** 10.1186/s42523-023-00248-6

**Published:** 2023-04-29

**Authors:** Juliana Botero, Atena Sadat Sombolestani, Margo Cnockaert, Charlotte Peeters, Wim Borremans, Luc De Vuyst, Nicolas J. Vereecken, Denis Michez, Guy Smagghe, German Bonilla-Rosso, Philipp Engel, Peter Vandamme

**Affiliations:** 1 Laboratory of Microbiology, Department of Biochemistry and Microbiology, Ghent University, K. L. Ledeganckstraat 35, 9000 Ghent, Belgium; 2 Research Group of Industrial Microbiology and Food Biotechnology, Department of Bioengineering Sciences, Faculty of Sciences and Bioengineering Sciences, Vrije Universiteit Brussel, Pleinlaan 2, 1050 Brussels, Belgium; 3 Agroecology Lab, Université libre de Bruxelles, Boulevard du Triomphe CP 264/02, 1050 Brussels, Belgium; 4 Laboratory of Zoology, Research Institute for Biosciences, University of Mons, Place du parc 20, 7000 Mons, Belgium; 5 Laboratory of Agrozoology, Department of Plants and Crops, Faculty of Bioscience Engineering, Ghent University, Coupure Links 653, 9000 Ghent, Belgium; 6 Department of Fundamental Microbiology, University of Lausanne, CH-1015 Lausanne, Switzerland

**Keywords:** *Commensalibacter*, Insect symbiont, Asian hornet, Bumble bee, Western honey bee, Butterfly, Phylogenomics, Functional genomics

## Abstract

**Background:**

To understand mechanisms of adaptation and plasticity of pollinators and other insects a better understanding of diversity and function of their key symbionts is required. *Commensalibacter* is a genus of acetic acid bacterial symbionts in the gut of honey bees and other insect species, yet little information is available on the diversity and function of *Commensalibacter* bacteria. In the present study, whole-genome sequences of 12 *Commensalibacter* isolates from bumble bees, butterflies, Asian hornets and rowan berries were determined, and publicly available genome assemblies of 14 *Commensalibacter* strains were used in a phylogenomic and comparative genomic analysis.

**Results:**

The phylogenomic analysis revealed that the 26 *Commensalibacter* isolates represented four species, i.e. *Commensalibacter intestini* and three novel species for which we propose the names *Commensalibacter melissae* sp. nov., *Commensalibacter communis* sp. nov. and *Commensalibacter papalotli* sp. nov. Comparative genomic analysis revealed that the four *Commensalibacter* species had similar genetic pathways for central metabolism characterized by a complete tricarboxylic acid cycle and pentose phosphate pathway, but their genomes differed in size, G + C content, amino acid metabolism and carbohydrate-utilizing enzymes. The reduced genome size, the large number of species-specific gene clusters, and the small number of gene clusters shared between *C. melissae* and other *Commensalibacter* species suggested a unique evolutionary process in *C. melissae*, the Western honey bee symbiont.

**Conclusion:**

The genus *Commensalibacter* is a widely distributed insect symbiont that consists of multiple species, each contributing in a species specific manner to the physiology of the holobiont host.

**Supplementary Information:**

The online version contains supplementary material available at 10.1186/s42523-023-00248-6.

## Background

Bee health is endangered by various factors including pesticide exposure, habitat loss, and elevated loads of parasites [1, 2]. Symbiotic gut microbiota of insects play essential roles in the health of their hosts, through mechanisms that include the suppression of pathogens, and therefore contribute to gut homeostasis and host fitness [3–5]. Symbiotic associations between bacteria of the *Acetobacteraceae* family and their insect hosts have received great attention, particularly in pollinators because of their key contribution to ecosystem functioning and their role in agricultural production [3]. The genera *Commensalibacter* and *Bombella* are acetic acid bacteria that belong to the *Acetobacteraceae* family and are regarded as non-core gut symbionts of honey bees because they are generalists that are also able to colonize other bee-associated environments such as beebread and honeycombs as well as the honey bee crop and gut [2, 4].

*Commensalibacter* bacteria have been detected in and isolated from the intestines of several insects that feed on high carbohydrate diets including honey bees (*Apis mellifera*, *Apis florea* and *Apis dorsata*) [2, 6–10], bumble bees (*Bombus hypnorum* and *Bombus pascuorum)* [11], small carpenter bees (*Ceratina calcarata*) [12], firebugs (*Probergrothius angolensis*) [13], and butterflies (*Heliconius* and several related genera) [14–17]. In honey bees, *Commensalibacter* has a particular caste association since it is more commonly found in larvae, drones and queen guts [18, 19], especially in early stages of gut microbiome colonization [20, 21] and Kesnerova et al. [2] reported an increase in relative abundance of *Commensalibacter* in long-lived winter bees. Today, only a single species, isolated from the gut of *Drosophila melanogaster*, has been formally named, i.e. *Commensalibacter intestini* [22, 23]. Additionally, strain MX01, an isolate from the gut of a monarch butterfly (*Danaus plexippus*) was shown to represent a novel *Commensalibacter* species that was tentatively named “*Commensalibacter papalotli*” [24], but this name has no standing in bacterial nomenclature [25]. Its whole-genome sequence, along with that of several honey bee isolates [26], is publicly available.

Little is known about the taxonomic and functional diversity of *Commensalibacter* isolates from different insect hosts, or its cohesiveness as a genus. Several reports suggest that *Commensalibacter* strains are associated with the health of their respective insect hosts. For example, *C. intestini* was reported to be involved in modulating *Drosophila* immunity and suppressing the proliferation of *Gluconobacter morbifer* by competition [3]. Similarly, Hubert et al. showed that the relative abundance of *Commensalibacter* was increased in adult honey bees infested with varroosis, a disease caused by mites [8]. Moreover, an increased abundance of *Commensalibacter* was correlated with longer host life span in *Speyeria mormonia* butterflies [17]. Finally, comparative genomic analyses of *Commensalibacter* and *Bombella* isolates from honey bees suggested that *Commensalibacter* has an advantage in the worker bee hindgut compared to *Bombella*, due to its broader metabolic range [26]. Despite their potential importance, the mechanistic understanding of these functional associations, especially considering the wide range of hosts which *Commensalibacter* can interact with, remains elusive.

In the present study we used comparative genomic analysis to revisit the taxonomy and functional potential of the genus *Commensalibacter*. This analysis includes the genomes of 14 publicly available honey bee [26, 27], fruit fly [28, 29] and butterfly isolates [24], complemented with draft genomes of 12 novel *Commensalibacter* isolates from bumble bees, rowan berries, hornets and butterflies.

## Methods

### *Commensalibacter* isolates and cultivation conditions

Novel *Commensalibacter* isolates were obtained in the course of several large-scale isolation campaigns in Belgium ([11, 30] and unpublished data), which involved the use of multiple isolation media and incubation conditions, and the application of MALDI-TOF mass spectrometry for isolate dereplication and preliminary identification [31, 32] (Table 1). This dereplication step allowed to limit the number of isolates for subsequent identification analyses, as isolates with distinct mass spectra are considered to represent genetically distinct strains [11, 32]. Twelve of these isolates were selected for whole genome sequence analysis in the present study (Table 1). Table 1 gives an overview of the *Commensalibacter* isolates obtained in these isolation campaigns, their sources, growth media and atmospheric conditions used for primary isolation, and the strain designation of the isolates selected on the basis of MALDI-TOF MS pattern diversity for whole-genome sequence analysis in the present study.

**Table 1:** Overview of new *Commensalibacter* isolates, their isolation source and other isolation details. All isolates originated from Belgium. Insect samples were whole gut samples

Source	N^1^	Isolation medium^2^	Isolation year	Strain
*Bombus pascuorum*	3	M13-A, M404-A	2013	LMG 28296
*Bombus hypnorum*	3	AC–MA	2013	LMG 31819^T^ and R-53529
*Vespa velutina* ^3^	12	M13–A, M404-A	2019	LMG 32512^T^
*Sorbus aucuparia* berries	12	MRS-A	2020	R-79671 to R-79674
*Aglais io*	1	TSA–A	2022	R-83493
*Vespa velutina* ^3^	3	M13–A	2022	R-83526
*Pieris rapae* ^4^	55	M13–A, M404-A	2022	R-83534
*Vespa velutina* ^3^	1	M13–A	2022	R-83540

^1^Number of isolates with indistinguishable MALDI-TOF mass spectra

^**2**^AC, all culture agar (20 g/l tryptose, 3 g/l beef extract, 3 g/l yeast extract, 3 g/l malt extract, 5 g/l dextrose, 0.2 g/l ascorbic acid, 15 g/l agar); M13, LMG medium 13 (25% g/l D-mannitol, 5 g/l yeast extract, 3 g/l bacteriological peptone, 15 g/l agar)

M404, LMG medium 404 (50 g/l D-glucose; 10 g/l yeast extract, 15 g/l agar); MRS, Man, Rogosa and Sharpe agar (Oxoid); TSA, Tryptone Soya Agar (Oxoid); A, aerobic incubation; MA, microaerobic incubation. All isolation media comprised 10 ppm cycloheximide (Sigma-Aldrich) to inhibit fungal growth

^*3*^LMG 32512^T^, R-83526 and R-83540 were isolated from different *V. velutina* hornets each

^4^Gut samples from two *P. rapae* butterflies were analyzed

*Commensalibacter* reference strains LMG 31900^T^ (= ESL0284^T^) and LMG 27436^T^ (= A911^T^) were obtained from the BCCM/LMG Bacteria Collection (Ghent, Belgium). The former was isolated from a Western honey bee gut sample in Switzerland [26]; the latter is the *C. intestini* type strain, and was isolated from a gut sample of *Drosophila melanogaster* in South Korea [23]. All strains were routinely cultivated on LMG agar medium 404 [50 g/l d-glucose, 10 g/l yeast extract, and 15 g/l agar) and incubated under aerobic conditions at 28 °C for 48 h.

### DNA extraction, sequence analysis and genome assembly

Genomic DNA of isolates LMG 28296, LMG 31819^T^, LMG 32512^T^, R-53529, R-79671, R-79672, R-79673 and R-79674 was extracted using the Maxwell Tissue DNA kit (Promega, USA) and the Maxwell RSC instrument according to the manufacturer’s instructions; genomic DNA of isolates R-83493, R-83526, R-83534, R-83540 was extracted using the Maxwell Cultured Cells DNA kit (Promega, USA). DNA extracts were treated with RNase (2 mg/mL, 5 µL per 100 µL of extract) and incubated at 37 °C for one hour. DNA quality was checked using 1% agarose gel electrophoresis and DNA quantification was performed using the QuantiFluor ONE dsDNA system and the Quantus fluorometer (Promega, USA). Whole-genome sequencing was carried out on the Illumina HiSeq 4000 (R-53529) or NovaSeq 6000 (LMG 28296, LMG 31819^T^, LMG 32512^T^, R-79671, R-79672, R-79673 and R-79674) platform at the Oxford Genomics Centre (Oxford, UK), or on the NextSeq 2000 platform (R-83534, R-83493, R-83526 and R-83540) at MiGS center (Pittsburgh, USA).

A quality check of the reads was performed using FastQC v0.11.9 (https://www.bioinformatics.babraham.ac.uk/projects/fastqc/) and the results were compiled into a single report by using MultiQC 1.9 [33]. Reads were filtered by removing low-quality sequences using fastp v0.20.1 [34] in simple usage. *De novo* assemblies were obtained with Shovill v1.0.4 (https://github.com/tseemann/shovill) [35] with disabled error correction and default settings. Contigs shorter than 500 bp were removed from the final assembly. Reads were mapped to the assemblies using BWA v0.7.17 [36] and the resulting summary statistics, including mapped reads and coverage, were calculated with SAMtools v1.11 [37] and Qualimap v2.2.1 [38]. PlasmidHunter was used for the identification of plasmids [39].

In addition, all 29 publicly available *Commensalibacter* genome assemblies and the genome assemblies of type strains representing additional acetic acid bacterial genera (Supplementary Tables S1 and S2 were downloaded from the NCBI database (June 3, 2022) by using the E-utilities Command [40]. CheckM v1.1.2 was used to estimate genome completeness and contamination [41]. The G + C content and genome size were calculated using QUAST v5.0.2 [42]. The 16S rRNA gene sequences were extracted from the draft genomes using the BAsic Rapid Ribosomal RNA Predictor software (Barrnap) (https://github.com/tseemann/barrnap) and were submitted to the EzBiocloud identification tool [43].

### Phylogenomic analyses

The whole-genome sequences of *Commensalibacter* isolates and of representative phylogenetic neighbors were used to construct a phylogenomic tree based on 107 single-copy genes using bcgTree [44] and a partitioned maximum-likelihood analysis in RAxML v8.2.12 [45]. Visualization and annotation of the tree were performed using iTOL [46]. To verify taxonomy, genomes were submitted to the Type Strain Genome Server (TYGS) [47], and digital DNA-DNA hybridization (dDDH, formula d4) values were calculated using the Genome-to-Genome Distance Calculator (GGDC 2.1) with recommended settings [48]. In addition, Average Nucleotide Identity (ANI) values were calculated by using the OrthoANIu algorithm using the standalone tool [49].

### Annotation and comparative genomic analyses

The Anvi’o pangenomics suite was used to perform annotation (COG and KEGG) and a comparative genomics analysis of *Commensalibacter* genomes. An MCL inflation parameter of 8 was used to assign the protein-coding DNA sequences (CDSs) to clusters of orthologous genes. CDS generated by anvi’o were also annotated using EggNOG-mapper v2.1.7 with the EggNOG database v5.0.2 [50, 51]. Based on COG [52] and KEGG orthology [53, 54], each CDS was assigned to its respective COG, COG category, KEGG, KEGG module, KEGG reaction and KEGG pathway. The COG and KEGG annotations obtained by both tools (i.e. anvi’o and EggNOG-mapper) were combined to obtain a higher proportion of annotated CDSs. Genus core genes and species core genes were inferred from the gene clusters. A gene cluster was considered to belong to the genus or species core if it was present in all the members of the group. COG annotation was used to assess defense mechanisms. KEGG annotation was used for the calculation of the KEGG module completeness fraction (mcf) by using the KO_mapper script from MicrobeAnnotator [55] and the KEGGREST was used to determine the reactions from the KEGG numbers for the identification of carbohydrate-utilizing enzymes. MacSyFinder v2 was used to identify bacterial secretion systems [56]. Finally, Virsorter2 [57] was used to identify prophages sequences and the resulting sequences were compared to each other using blast+ [58].

### Data analysis

Anvi’o results, the COG20 database, MicrobeAnnotator results and KEGG hierarchy were imported in R 4.1.3 and analyzed using tidyverse, imputeTS, matrixStats, fuzzyjoin, ggnewscale, ComplexHeatmap and ggven packages.

### Phenotypic tests

Cell morphology and phenotypic characteristics of *C. intestini* LMG 27436^T^ and *Commensalibacter* isolates LMG 31819^T^, LMG 32512^T^ and LMG 31900^T^ were examined as described before [59]. For testing growth in the presence of 1 and 2% NaCl and in the presence of 10% ethanol, standard medium (SM) [0.5% (m/v) yeast extract and 5% (m/v) d-glucose] was used. To test the growth on nitrate, all isolates were grown on trypticase soy agar (TSA, Oxoid) supplemented with 0.1% KNO_3_ and incubated for 7 days at 28 °C under anaerobic conditions. As a control experiment, isolates were also incubated on TSA without KNO_3_ and were incubated under the same conditions.

## Results and discussion

### Genome characteristics

The genome characteristics of all *Commensalibacter* isolates included in the present study were listed in Supplementary Table S2. The 12 new genomes resulted in assemblies of 16 to 85 contigs and genome sizes of 2.34 to 2.58 Mbp. CheckM analysis (with marker lineage *Rhodospirillales*) revealed more than 99% completeness and less than 0.75% contamination in each of these 12 draft assemblies. For ESL0284^T^, only one of the two publicly available assemblies was retained for further analysis. Only 14 of the publicly available *Commensalibacter* genomes comprised 16S rRNA gene sequences and therefore the remaining 14 *Commensalibacter* genome assemblies were excluded from further analysis.

A single copy of the rRNA operon was detected in each of the retained assemblies (Supplementary Table S2), except in *Commensalibacter* sp. ESL0284^T^ (GCF_009734185.1) and *Commensalibacter* sp. AMU001 (GCF_003691365.1), which are complete genome sequences, and both genome assemblies comprised four identical copies of the rRNA operon. Upon remapping reads of each of the 12 new genome assemblies, we noted that the depth of coverage of the rRNA operon was approximately four times that of the remainder of the genome, suggesting that these *Commensalibacter* genome assemblies comprised four identical copies of the rRNA operon. Plasmid sequences were detected in each of the 12 new *Commensalibacter* genomes. These sequences were present in 3 to 7 contigs in the assemblies (data not shown).

### Phylogenomic analyses

**Fig. 1:**
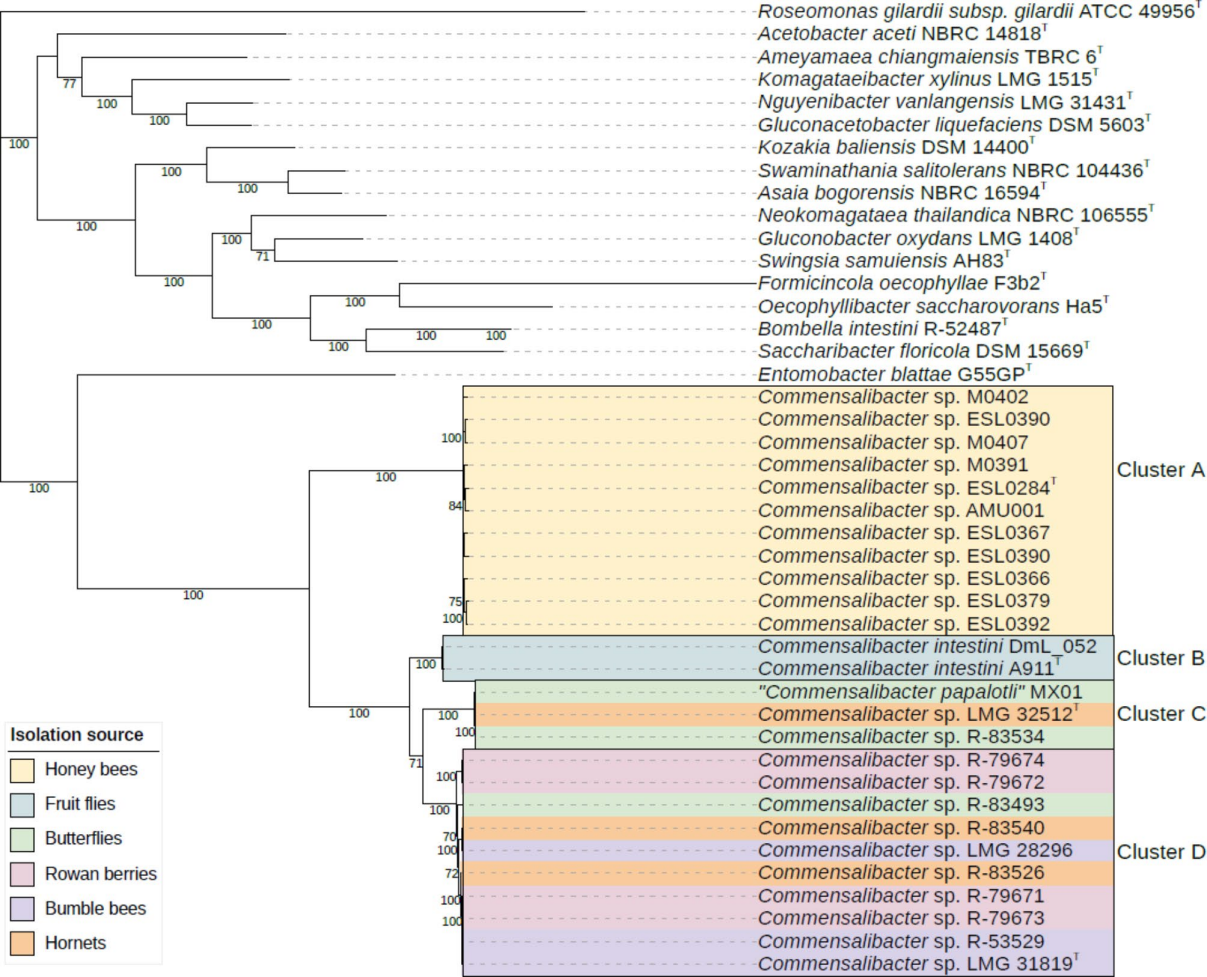
Maximum-likelihood bcgTree tree based on 107 core genes showing the phylogenomic relationships between the *Commensalibacter* and representative phylogenetic neighbor taxa. Bootstrap percentages above 70% (1000 replicates) are shown next to the branch points. The color bar indicates isolation sources. Superscript ‘T’ denotes taxonomic type strains

The phylogenomic analysis confirmed that all 26 *Commensalibacter* isolates represented a single line of descent within the acetic acid bacteria, with *Entomobacter blattae* as nearest neighbor taxon (Fig. 1). The *Commensalibacter* lineage comprised four well-separated clusters with high bootstrap support. A first cluster (cluster A) comprised *Commensalibacter* sp. ESL0284^T^ and ten additional honey bee isolate genomes. A second cluster (cluster B) comprised the two *C. intestini* isolate genomes. A third cluster (cluster C) comprised the “*C. papalotli*” MX01, the Asian hornet isolate *Commensalibacter* sp. LMG 32512^T^ and butterfly isolate *Commensalibacter* sp. R-83534 genomes. Finally, a fourth cluster (cluster D) comprised the genomes of three bumble bee isolates (LMG 28296, LMG 31819^T^ and R-53529), the rowan berry isolates (R-79671 through R-79674), two Asian hornet isolates (R-83526 and R-83540) and a single butterfly isolate (R-83493).

OrthoANIu and dDDH values were calculated between each pair of *Commensalibacter* genomes (Supplementary Fig. S1) and revealed that each of the four clusters represented a group of isolates sharing dDDH and orthoANIu values above the species delineation thresholds of 70% dDDH [48] and 95–96% orthoANIu [60]. In addition, dDDH and orthoANIu between isolates of different clusters were well below both species delineation thresholds (Supplementary Fig. S1). Together, these data demonstrated that the four clusters corresponded with four *Commensalibacter* species. Below, we formally propose the names *Commensalibacter melissae* sp. nov. for the cluster A isolates, *Commensalibacter papalotli* sp. nov. for all cluster C isolates and *Commensalibacter communis* sp. nov. for all cluster D isolates (Supplementary information).

Supplementary Fig. S2 presents the estimated G + C content and genome size of each of the genomes analyzed and revealed that the four *Commensalibacter* species were characterized by distinct G + C content and genome size ranges. *Commensalibacter melissae* genomes were characterized by the highest G + C content (37.67 ± 0.08 mol %) and the smallest genome sizes (1.99 ± 0.03 Mbp), suggesting a genomic reduction that may reflect features of their ecology and their specialized association with honey bees [61]. *Commensalibacter communis* genomes had a G + C content (37.40 ± 0.02 mol %) that was slightly lower than that of the *C. melissae* genomes, but had the largest genome sizes (2.51 ± 0.04 Mbp). In contrast, *C. papalotli* and *C. intestini* genomes were similar in size (2.35 ± 0.02 Mbp and 2.44 ± 0.01 Mbp, respectively) and had the lowest G + C content (36.68 ± 0.02 mol % and 36.83 ± 0.02 mol %, respectively).

### Comparative genomic analysis

**Fig. 2:**
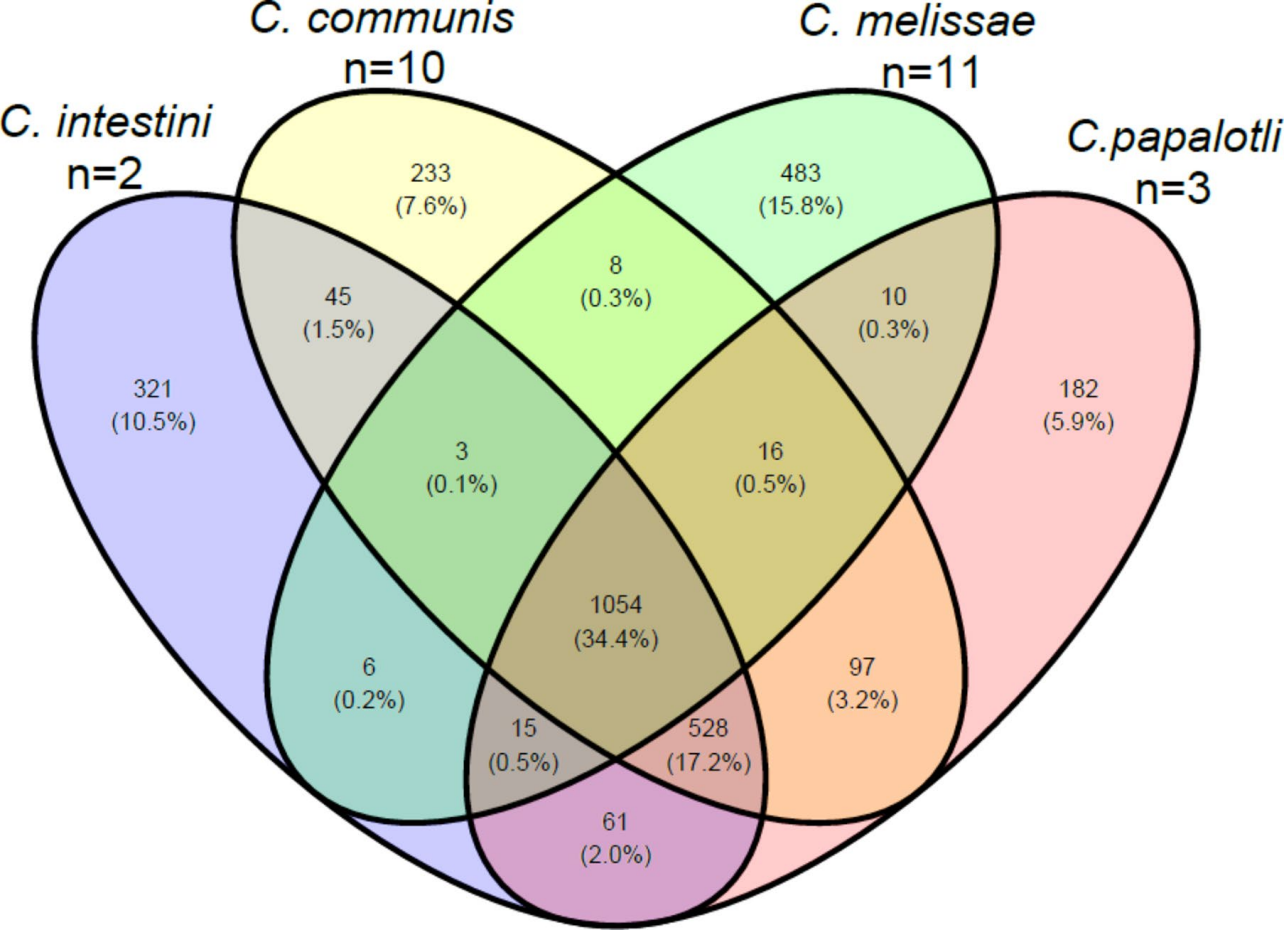
Venn diagram representing the species and genus core gene clusters within *Commensalibacter*. N is the number of genomes analyzed

**Fig. 3:**
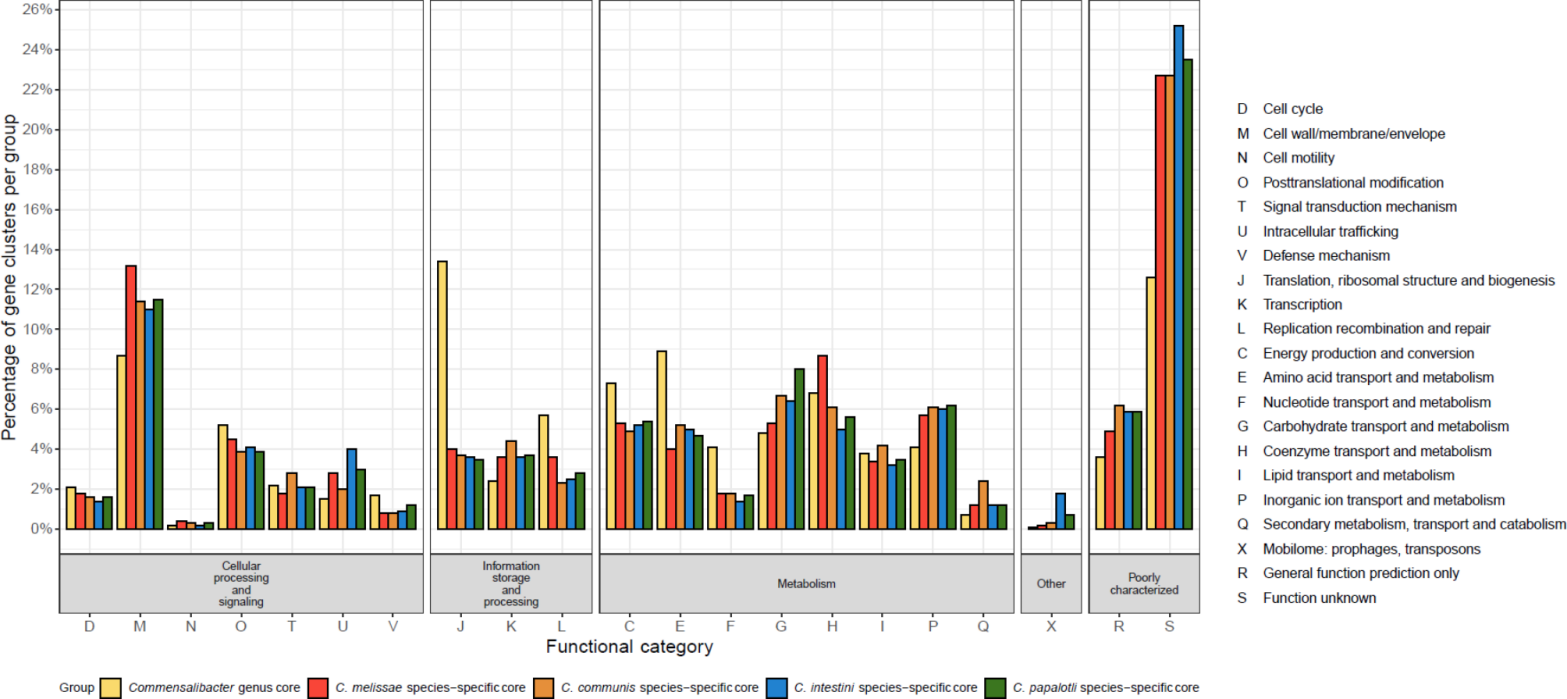
Distribution of gene clusters over COG categories of the *Commensalibacter* genus core and *Commensalibacter* species core genomes. Percentages of gene clusters per group are presented

The *Commensalibacter* pangenome consisted of 4,523 gene clusters (54,280 CDS) which included a genus core set of 1,054 gene clusters (30,040 CDS), and 1,219 gene clusters (9,899 CDS) that were part of the species cores (Fig. 2). A total of 4,153 (68%) and 2,954 (48%) gene clusters were assigned to COG categories and KEGG ortholog groups, respectively. The distribution of COG categories among the *Commensalibacter* genus core genome and the *Commensalibacter* species core genomes (Fig. 3) showed that gene clusters with unknown function (S) were the largest category and represented 12.6% of the *Commensalibacter* genus core genome, and between 22.7% and 25.2% of each of the *Commensalibacter* species core genomes.

The distribution of gene clusters among COG categories (Fig. 3) in each of the *Commensalibacter* species core genomes was fairly homogeneous, with the *C. melissae* core genome as the most aberrant one in which cell wall/membrane/envelope (M) (13.2%), coenzyme transport and metabolism (H) (8.7%) and replication recombination and repair (L) (3.6%) gene clusters were overrepresented, and in which carbohydrate transport metabolism (G) (5.3%) was underrepresented. However, the number of gene clusters classified in each of these categories was lower in *C. melissae* compared to other groups (Supplementary Fig. S3). The *C. communis* core genome was relatively rich in transcription (K) (4.4%), secondary metabolism (Q) (2.4%) and signal transduction mechanism (T) (2.8%), possibly suggesting a superior environmental adaptability, as category K genes contain many transcriptional regulators (Fig. 3 and Supplementary Fig. S3 ). The *C. papalotli* core genome appeared particularly enriched in carbohydrate transport metabolism (G) (6.7%) (Fig. 3). Finally, the *C. intestini* core genome was particularly enriched in intracellular trafficking and secretion (U) (5.9%) and mobilome (X) (1.8%). The latter category included prophage and transposase genes (Fig. 3 and Supplementary Fig. S3 ).

### Amino acid metabolism

**Fig. 4:**
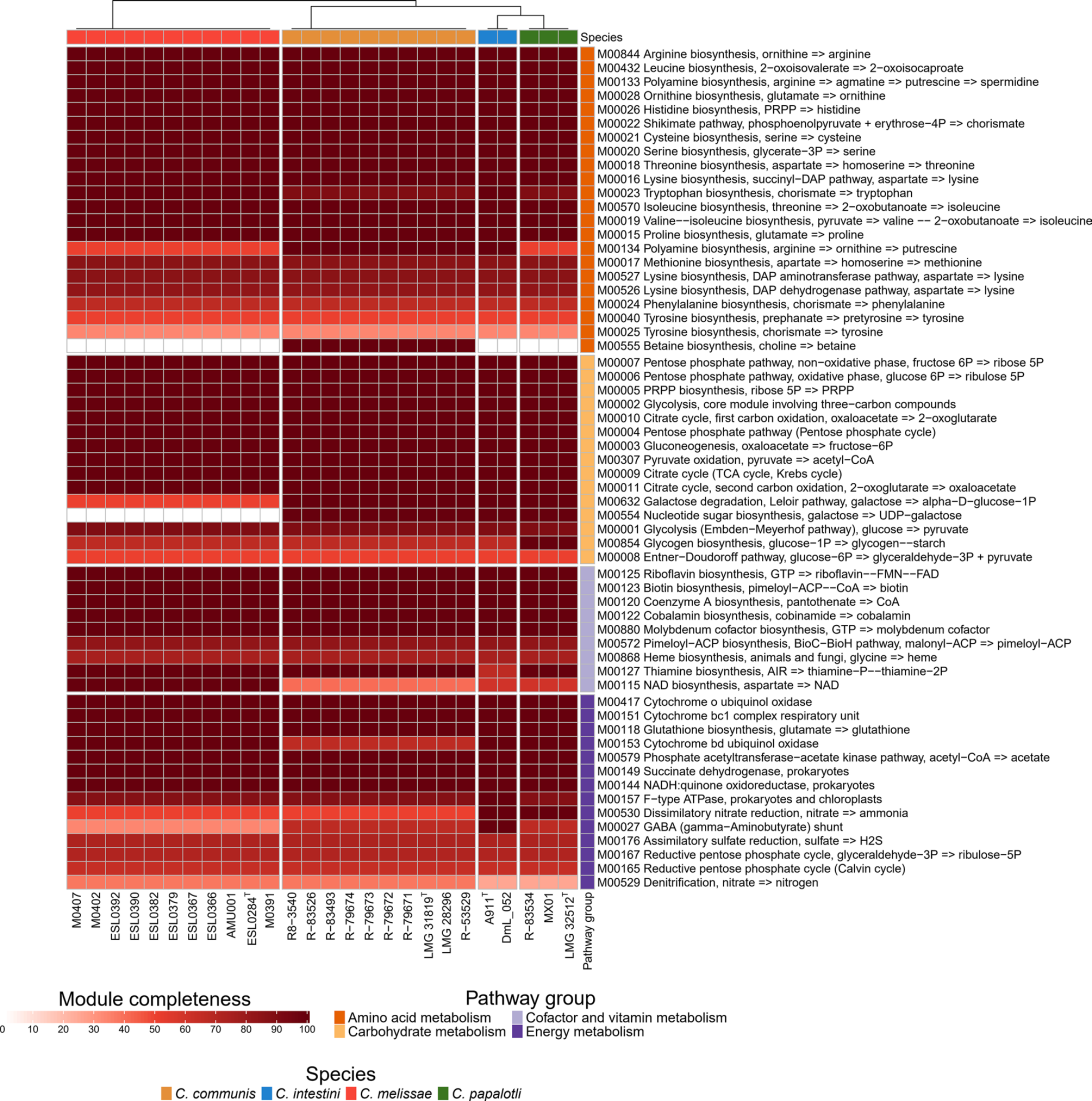
Heatmap illustrating the level of completeness of KEGG metabolic modules annotated by MicrobeAnnotator based on the presence and absence of genes. The color scale ranges from zero to 100 indicating the percentage of module completeness. Black and dark colors (the color scale is shown at the bottom) represent a complete or highly complete modules. while white and light colors refer to areas where a module is absent and highly incomplete. The dendrogram (at the top) shows that the species are clustered by the Pearson correlation

An analysis of the completeness of KEGG metabolic pathways revealed a species-specific occurrence and degree of completeness of various metabolic pathways (Fig. 4). Several insect symbionts have been reported to hydrolyze proteins and synthesize amino acids that are essential for proper growth and development of their insect host [62, 63]. *Commensalibacter* genomes encoded the biosynthesis of 11 amino acids, which included 8 out of 10 essential amino acids for bees (Fig. 4) [64, 65]. The pathway for methionine biosynthesis, which is an essential amino acid involved in the initiation of protein translation, was incomplete in the *Commensalibacter* genomes (M00017) because the *metB* gene was missing, as also reported in other insect symbionts with reduced genome sizes [66, 67]. It is unclear how these bacteria produce methionine. In addition, putrescine, a polyamine derived from the decarboxylation of ornithine (M00134), was exclusively encoded in *C. intestini* and *C. communis* genomes. The latter species was also capable of producing betaine (M00555), a powerful osmoprotectant that allows bacteria to survive and compete in environments with variable external osmolarity [68]. It has been suggested that nitrogenous waste products such as uric acid that are produced in the honey bee rectum may support persistence of *Commensalibacter* [20]. Uric acid degradation genes were detected in the *C. communis* genomes, but not in *C. melissae, C. papalotli* or *C. intestini* genomes. The former genomes encoded genes for the degradation of uric acid to ureidoglycine, which can be further converted into glycine (EC:1.14.13.113, EC:3.5.2.17, EC:4.1.1.97, EC: 3.5.2.5, EC: 3.5.3.9 and EC: 2.6.1.112.

### Carbohydrate metabolism

As in other acetic acid bacterial genomes [69], none of the *Commensalibacter* genomes encoded 6-phosphofructokinase which suggested that the Embden–Meyerhof–Parnas pathway (M00001) is non-functional (Fig. 4). In contrast, the oxidative and non-oxidative phases of the pentose phosphate pathway (M00006 and M00007) were complete, suggesting that this pathway is functional. The Entner–Doudoroff pathway is likely not functional (M00008) due to the absence of the enzyme that catalyzes 6-P-gluconate to D-glyceraldehyde-3-phosphate (EC: 4.1.2.14). All tricarboxylic acid cycle genes (M00009 and M00011) were present. Pathways for galactose catabolism (both M00554 and M00632) were complete only in *C. intestini*, *C. papalotli* and *C. communis* genomes.

An analysis of the presence of genes encoding 119 carbohydrate-utilizing enzymes and KEGG reactions belonging to carbohydrate metabolism again revealed a species-specific occurrence of these carbohydrate-utilizing enzymes (Supplementary Fig. S4). Genes related to 14 carbohydrate-utilizing enzymes were identified in *Commensalibacter* genomes. Glucose and fructose are the main carbohydrates in nectar, and hence in pollinator diets [70]. Fructose-utilizing enzymes were abundantly encoded in each of the genomes analyzed, but genes encoding glucose-utilizing enzymes were largely lacking in *C. melissae*, as were many of the other carbohydrate-utilizing enzymes. Indeed, some *Commensalibacter* genomes encoded enzymes that utilize less common carbohydrates such as mannose, lactose, arabinose, and melibiose, which are indigestible or toxic to many pollinators [71–73]. The detection of these genes suggests that *Commensalibacter* symbionts might mitigate effects induced by such carbohydrates [4, 74]. Furthermore, the presence of β-glucosidase (GH3) and β-mannanase (GH26) which hydrolyze glycosidic bonds in complex gluco- or manno-configured polysaccharides [75] such as hemicellulose polymers [76], suggested that *Commensalibacter* participates with other symbionts in the digestion of polysaccharides [77].

### Energy metabolism

Acetic acid bacteria have an unusual metabolism that is characterized by the oxidization of carbohydrates, sugar alcohols and ethanol to produce the corresponding sugar acids or acetic acid, a process executed by primary dehydrogenases located on the periplasmic side of the cytoplasmic membrane [78]. Such oxidation reactions are referred to as ‘oxidative fermentation’ reactions because they result in incomplete oxidation of compounds, which can eventually be further assimilated -or overoxidized- in a later growth phase [79, 80]. However, *Commensalibacter* bacteria can utilize a tricarboxylic acid cycle coupled to oxidative phosphorylation, which is energetically more efficient (Fig. 4). The 16 dehydrogenases/reductases reported by Bonilla-Rosso et al. [26] in honey bee *Commensalibacter* isolate genomes were also detected in the present study except for some slight differences. All *Commensalibacter* isolate genomes shared five dehydrogenases/reductases: three were able to oxidize metabolites (i.e. D-lactate dehydrogenase [EC 1.1.2.5], putative membrane-bound dehydrogenase [EC:1.5.5.1] and bifunctional proline dehydrogenase [EC 1.5.5.2]), cytochrome bc1 that transfers electrons from quinol to a higher potential acceptor protein (complex III), and cytochrome o ubiquinol oxidase that is the terminal electron acceptor oxidase (complex IV). The entire cytochrome bd ubiquinol gene complex (alternative complex IV) was detected in *C. intestini*, *C. papalotli* and *C. melissae* genomes. Moreover, *C. communis* and *C. papalotli* genomes all encoded the same dihydro-orotate dehydrogenase (DHOD), where a different DHOD was detected in *C. melissae* genomes. In total, five dehydrogenases/reductases were specific to *Commensalibacter* (four of which could oxidize succinate, NADH, glycerol 3-phosphate and malate, and one of which could reduce nitrate).

Strikingly, all *Commensalibacter* genomes encoded nitrate reductase (EC: 1.7.5.1), suggesting the capacity to gain energy through anaerobic respiration. Only *C. intestini* and *C. papalotli* genomes encoded the complete pathway for dissimilatory nitrate reduction to ammonia (M00530, Fig. 4), where *C. melissae* and *C. communis* genomes encoded the conversion of nitrate to nitrite, and nitric oxide to nitrous oxide (nitric oxide reductase norB gene, EC: 1.7.2.5). When inoculated on TSA supplemented with 0.1% KNO_3_ and incubated anaerobically, growth was observed in *C. papalotli*, weak growth was noted in *C. intestini* and *C. communis*, and no growth was observed in *C. melissae* (data not shown). In the microaerobic environment of an insect gut where oxygen remains the main electron acceptor, it is likely that nitric oxide reductase has a detoxifying role [81].

### Interactions with host cells and other gut microorganisms

*Commensalibacte*r genomes contained ~ 14 defense mechanism gene clusters (Supplementary Fig. S5). Type 1 and type 5 secretion systems, multidrug efflux pump genes (COG1132 and COG2076) and a bacteriocin exporter gene (COG2274) were present in all genomes studied, where CRISPR-cas genes were uniformly absent, as previously reported [26]. Other defense mechanism genes were occasionally detected, but not in a species-specific manner [82, 83]. These include genes related to type I (COG0610) and type II (COG1002) restriction-modification systems [84] and toxin-antitoxin systems (i.e. YeeF-YezG and RelBE) [85]. In addition, *C. melissae* genomes carried genes for the detoxification of formaldehyde by catalyzing S-formylglutathione into formate (COG0627). Formaldehyde is highly toxic to animals and bacteria, but can be detoxified by some organisms [86]. The presence of formaldehyde detoxification genes may suggest that formaldehyde can be produced by the host or by other host microbiota as a defense mechanism, as reported during the *Varroa destructor* infection process in honey bees [87], for which the *Commensalibacter* relative abundance increased by increasing varroosis levels [8].

COG category X comprised some prophage-associated genes, particularly in the *C. intestini* and *C. papalotli* genomes (Fig. 3), and multiple prophage sequences were detected using Virsorter2 [57]: between one and five in *C. melissae*, between one and nine in *C. communis*, between four and 15 in *C. papalotli*, and six each in *C. intestini* genomes. Prophage sequences that occurred in multiple genomes were consistently 100% identical within species, but differed between species (data not shown), except for the *C. papalotli* LMG 32512^T^ genome which comprised prophage sequences that were 100% identical and with more than 90% of query coverage, towards prophage sequences observed in the *C. communis* R-79673, R-79671, R-53529 and LMG 31819^T^ genomes.

Finally, all *Commensalibacte*r genomes encoded genes for the production of biotin (vitamin H, M00123), riboflavin (vitamin B2, M00125), niacin (vitamin B3, EC:6.3.4.21), pantothenic acid (vitamin B5, EC:6.3.2.1), pyrodoxal 5-phosphate, pyridoxal and pyridoxine (vitamin B6, EC:1.1.1.65 and EC:4.3.3.6), and cobalamin (vitamin B12, M00122) (Fig. 4). In addition, the thiamine (vitamin B1) biosynthesis pathway was completely encoded in the *C. melissae*, *C. communis*, and *C. papalotli*, but not the *C. intestini*, genomes. We failed to detect the *folA* gene and therefore could not confirm that *Commensalibacter* genomes encode folic acid biosynthesis (vitamin B9, M00126, M00841 and M00840) [26].

### Commensalibacter is a widely distributed insect symbiont

*Commensalibacter* bacteria are increasingly reported as members of the gut microbiota of pollinator and other insect species [2, 6–17]. Here, we analyzed genome sequences of a representative selection of new *Commensalibacter* isolates, along with publicly available reference strains and genome sequences. Together, the genus *Commensalibacter* was composed of four taxonomically and functionally different species (Figs. 1, 2, 3 and 4, Supplementary Figs. S1–S5) [22–24, 26, 27].

We hypothesized that the detection of two *Commensalibacter* species in Asian hornet samples reflected its predatory behavior on other insect species. We revisited publicly available 16S rRNA amplicon sequencing data reported in an Italian study of *V. velutina* hornets of different castes, life stages and colonies as well as colony samples [88]. Where the authors did not report or discuss *Commensalibacter* sequences in their study, a reanalysis of their amplicon sequencing variants (ASVs) revealed the presence of nine *Commensalibacter* ASVs (Supplementary Table S3) in their data set. These ASVs were detected in low abundances (< 1%) in workers, gynes, larvae and nest paper, thus supporting a hypothesis of non-colonizing bacteria that are in transit. However, in meconium samples an abundance of 13% was detected. Four ASVs were 100% identical to genome-derived 16S rRNA gene sequences of *C. melissae* ESL0284^T^, *C. communis* LMG 31819^T^, *C. papalotli* LMG 32512^T^, and *C. intestini* A911^T^. The remaining ASVs highlighted additional *Commensalibacter* sequence diversity, suggesting predation on insect hosts that carried other, hitherto unreported, *Commensalibacter* species. To the best of our knowledge, the isolation of *C. communis* from rowan berry samples in a small-scale study of acetic acid bacteria in fruit samples (unpublished data), represented the first report of environmentally isolated *Commensalibacter* bacteria. Although it is unclear where microbiota that are shared between flowers and pollinator species originate from, it is well-known that flowers are hubs of microbial transmission [89] and a study in Belgium showed that the *Sorbus* group is an important food source for bumble bees in anthropogenic environments [90].

### The taxonomic and functional diversity within other bee symbiont genera is poorly understood

The microbiota of honey bees and other eusocial corbiculate bees have been studied intensely, not only because these pollinators fulfill critical roles in ecosystem services and agriculture, but also because the bee microbiome serves as a model for evolution and ecology of host-microbe interactions [4, 91, 92]. While the honey bee microbiome in particular is simple and highly conserved there is misconception in literature regarding its taxonomic complexity. Five core phylotypes -or ‘species’- have consistently been reported, along with considerable strain-level variation within each of the core species [93–96]. In most of these phylotypes sequence discrete populations have now been observed, some of which corresponded with named species [95, 96]. While it has become gradually clear that the five core phylotypes in the three major corbiculate bee clades, i.e. *Apis, Bombus* and stingless bee species, correspond more with named bacterial genera rather than with single named species, authors continue to treat phylotypes and species as synonymous terms [92, 97]. From a taxonomic perspective, the five core phylotypes correspond with the genera *Snodgrassella* (the so-called Beta phylotype [98]), *Gilliamella* (Gamma-1), *Lactobacillus* (Firm-5), *Bombilactobacillus* (Firm-4) and *Bifidobacterium* (Bifido-1 and Bifido-2), while the *Bartonella* (Alpha-1), *Commensalibacter* (Alpha 2.1), *Bombella* (Alpha 2.2), *Frischella* (Gamma-2), *Apilactobacillus* (Lacto-3), *Bombiscardovia* (Bifido), and *Apibacter* (Bacteroides) phylotypes are considered non-core bacteria [2, 4].

The functional potential of these symbionts is gradually being explored. *Bifidobacterium* and *Gilliamella* are considered primary degraders of hemicellulose [77]. The genomes of these two bacteria, along with *Snodgrassella*, *Bartonella*, *Lactobacillus*, and *Bombilactobacillus*, encode genes that catalyze the reactions of a wide variety of polysaccharides and monosaccharides, including pectin-degrading enzymes and glycoside hydrolases, and therefore have the potential to aid in the breakdown of pollen, the release of nutrient-rich components thereof, and the removal of toxic sugars [99, 100]. In contrast, *Apibacter* [101, 102] and *Bombella* [103, 104] genomes mainly encode enzymes for the utilization of simple mono-saccharides or sucrose. Bee symbionts also have diverging capacities for the biosynthesis of amino acids and other vitamins. *Gilliamella*, *Snodgrassella*, *Apibacter*, *Bifidobacterium, Bartonella* and *Bombella* genomes encode the genes required for the synthesis of at least 18 amino acids; in contrast, *Bombilactobacillus* and *Lactobacillus* genomes present genes for the biosynthesis of some five amino acids [77, 101, 105]. Furthermore *Snodgrassella*, *Frischella*, and *Gilliamella* genomes encode genes for the biosynthesis of vitamins B2 and B9, and thiamine [106]; *Bombella* genomes comprise complete gene sets for the biosynthesis of vitamins B2, B3, B5, B6 and B9 [26]; *Bifidobacterium* genomes encode genes for the production of vitamins B6 and B9 [107], and *Apibacter* genomes contained genes involved in the biosynthesis of vitamin B2 only [102, 108].

While many other functional capacities and differences have been reported [97, 99, 101, 109, 110], very few comparative genomic or physiological studies systematically addressed functional differences between all species of a single symbiont genus, as in the present study [111–114]. The metabolic repertoire of a bacterial species is encoded in a core genome that is conserved within species and that typically comprises 75 to 90% of the gene content of any strain therein, and in an accessory genome that is strain specific [115], and functional analyses of bee symbionts are therefore best modeled on state-of-the art taxonomic information. The number of named species in each of the bee symbiont genera ranges now from two (*Frischella* and *Apibacter*) to 14 (*Bifidobacterium*) (Supplementary Table S4), and the metabolic capacities and differences of most of these species are yet to be explored through comparative genomic or physiological studies. While some of these species are clearly host-specific [97, 110, 116], many others co-occur in a single host [96]. The observation that genetically distinct but closely related strains partition their environment at fine phylogenetic and phylogenomic scales is not well understood [117]. The existence and function of such microdiversity, i.e. the co-occurrence of closely related but ecologically and physiologically distinct taxonomic groups, has been documented for about two decades and is an intrinsic property of many microorganisms [118]. A deeper mining of the genomic and physiological potential of different species of each of these bee symbiont genera will be required to improve our understanding of their functional roles and differences.

## Conclusion

The present study demonstrated that the genus *Commensalibacter* comprises at least four insect-associated species. Comparative genomic analyses revealed that the four *Commensalibacter* species had a similar genomic potential for central metabolism that was characterized by complete tricarboxylic acid cycle and pentose phosphate pathways, but their genomes differed in size, G + C content, and amino acid metabolism and carbohydrate-utilizing enzyme repertoires. *Commensalibacter melissae* genomes were most reduced in size. This was reflected in the loss of several metabolic pathways and even in pathways that encode metabolism of D-glucose, a key component of nectar (Fig. 4 and Supplementary Fig. S4). In concert with this, *C. melissae* genomes comprised the largest number of species-specific gene clusters and shared very few (10 or less) (Fig. 2) gene clusters with each of the three remaining *Commensalibacter* species. In contrast, *C. communis, C. papalotli*, and *C. intestini* shared 528 gene clusters which were absent in *C. melissae* (Fig. 2). There were clear metabolic differences between the former three species as well. *Commensalibacter communis* and *C. intestini* encoded the biosynthesis of putrescine, a commonly produced microbial metabolite that regulates multiple biological processes in the large intestine of humans and mice [119]. *Commensalibacter communis* was also capable of producing betaine, a powerful osmoprotectant that allows bacteria to survive and compete in environments with variable external osmolarity [68]. In particular the distribution of some carbohydrate-utilizing enzymes (i.e. D-galactose, xylitol, L-sorbose, D-xylose, D-mannose, L-rhamnose, lactose, L-arabinose, D-tagatose, D-galacturonic acid, L-ribulose, and L-xylulose) (Supplementary Fig. S4) differed markedly between these three species, and likely revealed a potential for detoxification or revealed cross-feeding mechanisms [74, 120]. Together, the reduced genome size, the large number of species-specific gene clusters, and the small number of gene clusters shared between *C. melissae* and other *Commensalibacter* species suggested a unique evolutionary process in *C. melissae*, the Western honey bee symbiont.

## Electronic supplementary material

Below is the link to the electronic supplementary material.

Supplementary Material 1

Supplementary Material 2

## Data Availability

The annotated genome assemblies were submitted to the European Nucleotide Archive (ENA) and are publicly available under project PRJEB54578.
