# Supplementary material for: A phylogenomic and comparative genomic analysis of Commensalibacter, a versatile insect symbiont
Source: Anim Microbiome. 2023 Apr 29;5:25. doi: 10.1186/s42523-023-00248-6 (PMC10149009; doi:10.1186/s42523-023-00248-6)
Supplement: Supplementary file 1 — Supplementary Material 1 [file 42523_2023_248_MOESM1_ESM.docx]

A phylogenomic and comparative genomic analysis of *Commensalibacter,* a versatile insect symbiont

Juliana Botero^1^, Atena Sadat Sombolestani^1^, Margo Cnockaert^1^, Charlotte Peeters^1^, Wim Borremans^2^, Luc De Vuyst^2^, Nicolas J. Vereecken^3^, Denis Michez^4^, Guy Smagghe^5^, German Bonilla-Rosso^6^, Philipp Engel^6^ and Peter Vandamme^1*^

**Supplementary information**

### Supplementary Table S1. Genome sequences used to construct a phylogenomic tree based on 107 single-copy genes.

| **Species** | **Strain** | **Assembly accession number** |
| --- | --- | --- |
| *Acetobacter aceti* | NBRC 14818^T^ | GCF_000193495.2 |
| *Bombella intestini* | R-52487^T^ | GCF_002003665.1 |
| *Ameyamaea chiangmaiensis* | BRC 6^T^ | GCF_018335475.1 |
| *Asaia bogorensis* | NBRC 16594^T^ | GCF_001547995.1 |
| *Entomobacter blattae* | G55GP^T^ | GCF_014672835.1 |
| *Formicincola oecophyllae* | F3b2^T^ | GCF_006542395.2 |
| *Gluconacetobacter liquefaciens* | DSM 5603^T^ | GCF_003350405.1 |
| *Gluconobacter oxydans* | LMG 1408^T^ | GCF_015244575.1 |
| *Komagataeibacter xylinus* | LMG 1515^T^ | GCF_003207915.1 |
| *Kozakia baliensis* | DSM 14400^T^ | GCF_001787335.1 |
| *Neokomagataea thailandica* | NBRC 106555^T^ | GCF_001598495.1 |
| *Nguyenibacter vanlangensis* | LMG 31431^T^ | GCF_013376155.1 |
| *Oecophyllibacter saccharovorans* | Ha5^T^ | GCF_006542375.1 |
| *Roseomonas gilardii subsp. gilardii* | A^T^CC 49956^T^ | GCF_023078375.1 |
| *Saccharibacter floricola* | DSM 15669^T^ | GCF_000378165.1 |
| *Swaminathania salitolerans* | NBRC 104436^T^ | GCF_007988945.1 |
| *Swingsia samuiensis* | AH83^T^ | GCF_006542355.1 |

### Supplementary Table S2. Genome characteristics of isolates included in the present study.

| **Isolate** | **Accession number** | **No. of contigs** | **Total length (bp)** | **G + C (%)** | **N50** | **N75** | **Completeness** | **Contamination** | **Coverage** | **No. Of CDSs** | **Isolation source** | **Country** |
| --- | --- | --- | --- | --- | --- | --- | --- | --- | --- | --- | --- | --- |
| ***C. intestini* A911^T^** | GCF_000231445.1 | 26 | 2,454,778 | 36.85 | 476,138 | 459,781 | 100 | 0.5 |  | 2260 | Fruit fly (*Drosophila melanogaster*) | South Korea |
| ***C. intestini* DmL_052** | GCF_002153535.1 | 50 | 2,434,976 | 36.81 | 278,278 | 146,194 | 99.5 | 0.5 |  | 2241 | Fruit fly (*Drosophila melanogaster*) | USA |
| **“*C. papalotli”* MX01** | GCF_000527695.1 | 12 | 2,332,652 | 36.66 | 1,547,573 | 415,471 | 99 | 0.75 |  | 2103 | Monarch butterfly (*Danaus plexippus*) | Mexico |
| ***Commensalibacter* sp.** |  |  |  |  |  |  |  |  |  |  |  |  |
| **LMG 28296** | GCA_947179055.1 | 41 | 2,499,938 | 37.38 | 447,223 | 117,992 | 99 | 0.75 | 270 | 2356 | Bumble bee (*Bombus pascuorum*) | Belgium |
| **LMG 31819^T^** | GCA_947179015.1 | 66 | 2,576,009 | 37.40 | 411,011 | 121,784 | 99 | 0.75 | 419 | 2472 | Bumble bee (*Bombus hypnorum*) | Belgium |
| **LMG 32512^T^** | GCA_947179115.1 | 48 | 2,381,376 | 36.71 | 356,792 | 207,381 | 100 | 0.75 | 359 | 2196 | Hornet (*Vespa velutina*) | Belgium |
| **R-53529** | GCA_947179095.1 | 85 | 2,581,439 | 37.42 | 410,971 | 117,267 | 99 | 0.75 | 445 | 2492 | Bumble bee (*Bombus hypnorum*) | Belgium |
| **R-79671** | GCA_947179065.1 | 43 | 2,528,666 | 37.45 | 446,462 | 116,700 | 99.5 | 0.75 | 615 | 2395 | *Sorbus aucuparia* berries | Belgium |
| **R-79672** | GCA_947179365.1 | 55 | 2,507,714 | 37.40 | 435,736 | 115,459 | 99.5 | 0.25 | 663 | 2392 | *Sorbus aucuparia* berries | Belgium |
| **R-79673** | GCA_947179045.1 | 39 | 2,528,503 | 37.44 | 446,462 | 198,306 | 99.5 | 0.75 | 659 | 2398 | *Sorbus aucuparia* berries | Belgium |
| **R-79674** | GCA_947179025.1 | 48 | 2,506,979 | 37.40 | 441,505 | 115,459 | 99.5 | 0.25 | 726 | 2395 | *Sorbus aucuparia* berries | Belgium |
| **R-83493** | GCA_947179105.1 | 31 | 2,421,906 | 37.41 | 432,330 | 136,560 | 99.5 | 0.75 | 171 | 2269 | European peacock (Butterfly *Aglais io*) | Belgium |
| **R-83526** | GCA_947179085.1 | 26 | 2,449,602 | 37.37 | 589,905 | 130,071 | 99.5 | 0.75 | 163 | 2284 | Hornet (Vespa velutina) | Belgium |
| **R-83534** | GCA_947179125.1 | 16 | 2,342,687 | 36.69 | 638,951 | 452,638 | 100 | 0.75 | 251 | 2105 | Cabbage butterfly (Pieris rapae) | Belgium |
| **R-83540** | GCA_947179075.1 | 50 | 2,513,132 | 37.36 | 301,298 | 192,473 | 99.5 | 0.75 | 226 | 2375 | Hornet (*Vespa velutina*) | Belgium |
| **AMU001** | GCF_003691365.1 | 2 | 2,023,834 | 37.83 | 2,013,174 | 2,013,174 | 99 | 0.75 |  | 1779 | Western honey bee (*Apis mellifera*) | Seychelles |
| **ESL0284^T^** | GCF_009734185.1 | 1 | 1,960,656 | 37.73 | 1,960,656 | 1,960,656 | 99 | 0.5 |  | 1701 | Western honey bee (*Apis mellifera*) | Switzerland |
| **ESL0366** | GCF_009725885.1 | 17 | 2,024,146 | 37.60 | 330,693 | 148,165 | 99 | 0.5 |  | 1823 | Western honey bee (*Apis mellifera*) | Switzerland |
| **ESL0367** | GCF_009725825.1 | 19 | 1,999,809 | 37.54 | 310,585 | 148,349 | 99 | 0.5 |  | 1775 | Western honey bee (*Apis mellifera*) | Switzerland |
| **ESL0379** | GCF_009725835.1 | 22 | 2,055,536 | 37.74 | 311,430 | 148,287 | 99.5 | 0.75 |  | 1863 | Western honey bee (*Apis mellifera*) | Switzerland |
| **ESL0382** | GCF_009725775.1 | 15 | 1,969,038 | 37.64 | 304,594 | 146,825 | 99 | 0.5 |  | 1732 | Western honey bee (*Apis mellifera*) | Switzerland |
| **ESL0390** | GCF_009725725.1 | 19 | 1,988,679 | 37.65 | 203,912 | 147,918 | 99 | 0.75 |  | 1793 | Western honey bee (*Apis mellifera*) | Switzerland |
| **ESL0392** | GCF_009725705.1 | 20 | 2,020,474 | 37.60 | 313,896 | 146,989 | 99 | 0.5 |  | 1820 | Western honey bee (*Apis mellifera*) | Switzerland |
| **M0391** | GCA_016102145.1 | 12 | 1,959,596 | 37.70 | 1,250,652 | 351,004 | 99 | 0.5 |  | 1724 | Western honey bee (*Apis mellifera*) | China |
| **M0402** | GCA_016101335.1 | 17 | 2,013,651 | 37.66 | 489,519 | 352,020 | 99 | 0.5 |  | 1797 | Western honey bee (*Apis mellifera*) | China |
| **M0407** | GCA_016101515.1 | 16 | 1,967,347 | 37.77 | 1,249,722 | 351,931 | 99 | 0.5 |  | 1740 | Western honey bee (*Apis mellifera*) | China |

### Supplementary Table S3. Percentage of identity between 16S rRNA sequences of the four *Commensalibacter* type strains and the 9 AVSs that were taken from Cini et al. [51].

|  | ASV1 | ASV2 | ASV3 | ASV4 | ASV5 | ASV6 | ASV7 | ASV8 | ASV9 |
| --- | --- | --- | --- | --- | --- | --- | --- | --- | --- |
| *C. melissae* ESL0284^T^ | 99.751 | 100 | 95.025 | 99.751 | 95.522 | 97.264 | 95.025 | 99.518 | 99.759 |
| *C. communis* LMG 31819^T^ | 94.776 | 95.025 | 100 | 95.274 | 98.259 | 97.761 | 99.005 | 94.699 | 94.94 |
| *C. intestini* A911^T^ | 94.776 | 95.025 | 99.005 | 95.274 | 97.264 | 96.766 | 100 | 94.699 | 94.94 |
| *C. papalotli* LMG 32512^T^ | 95.274 | 95.522 | 98.259 | 95.771 | 100 | 97.761 | 97.264 | 95.181 | 95.422 |


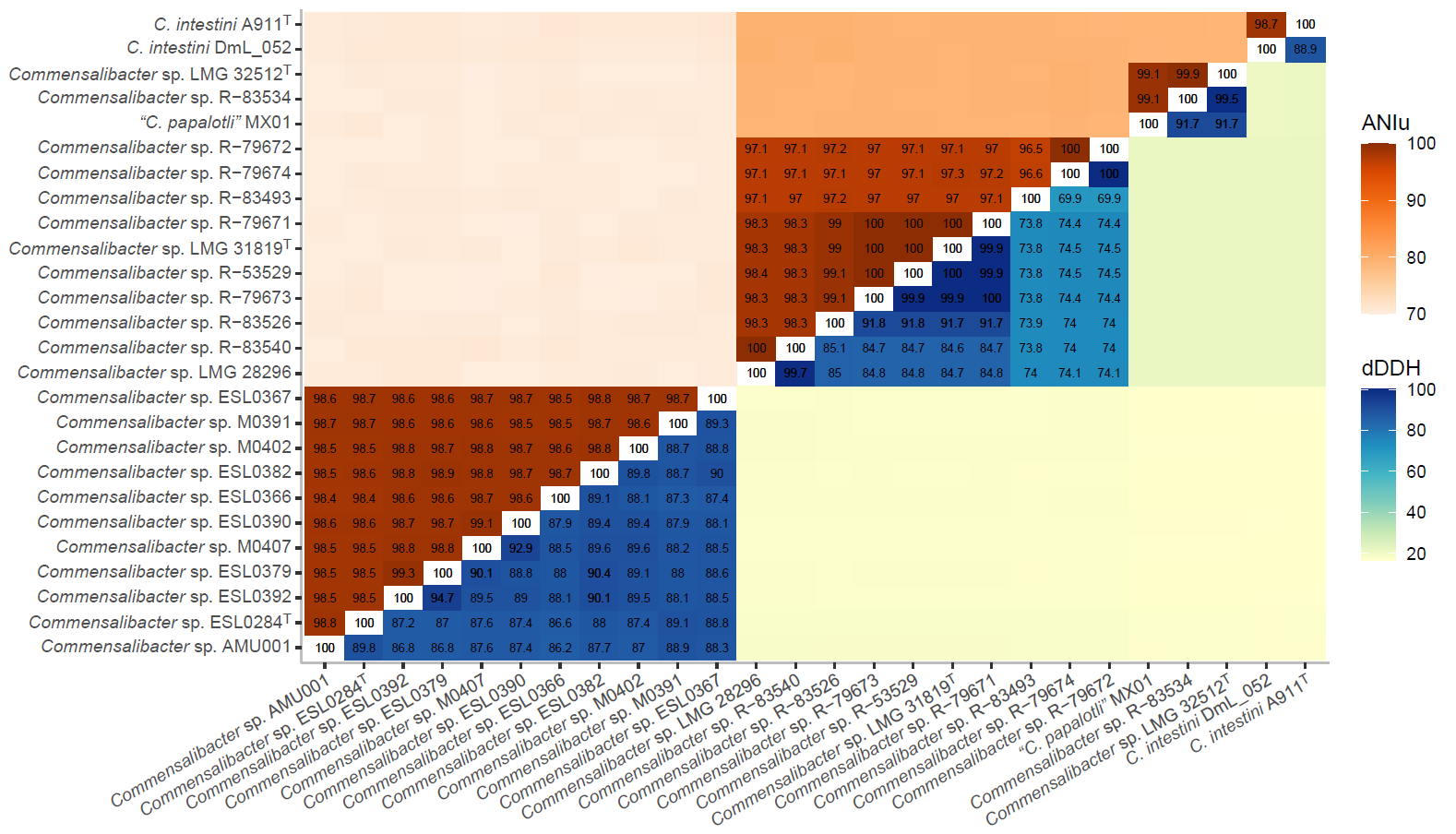


### Supplementary Figure S1. Heatmap displaying OrthoANIu (upper left) and dDDH (lower right) values between *Commensalibacter* genomes.


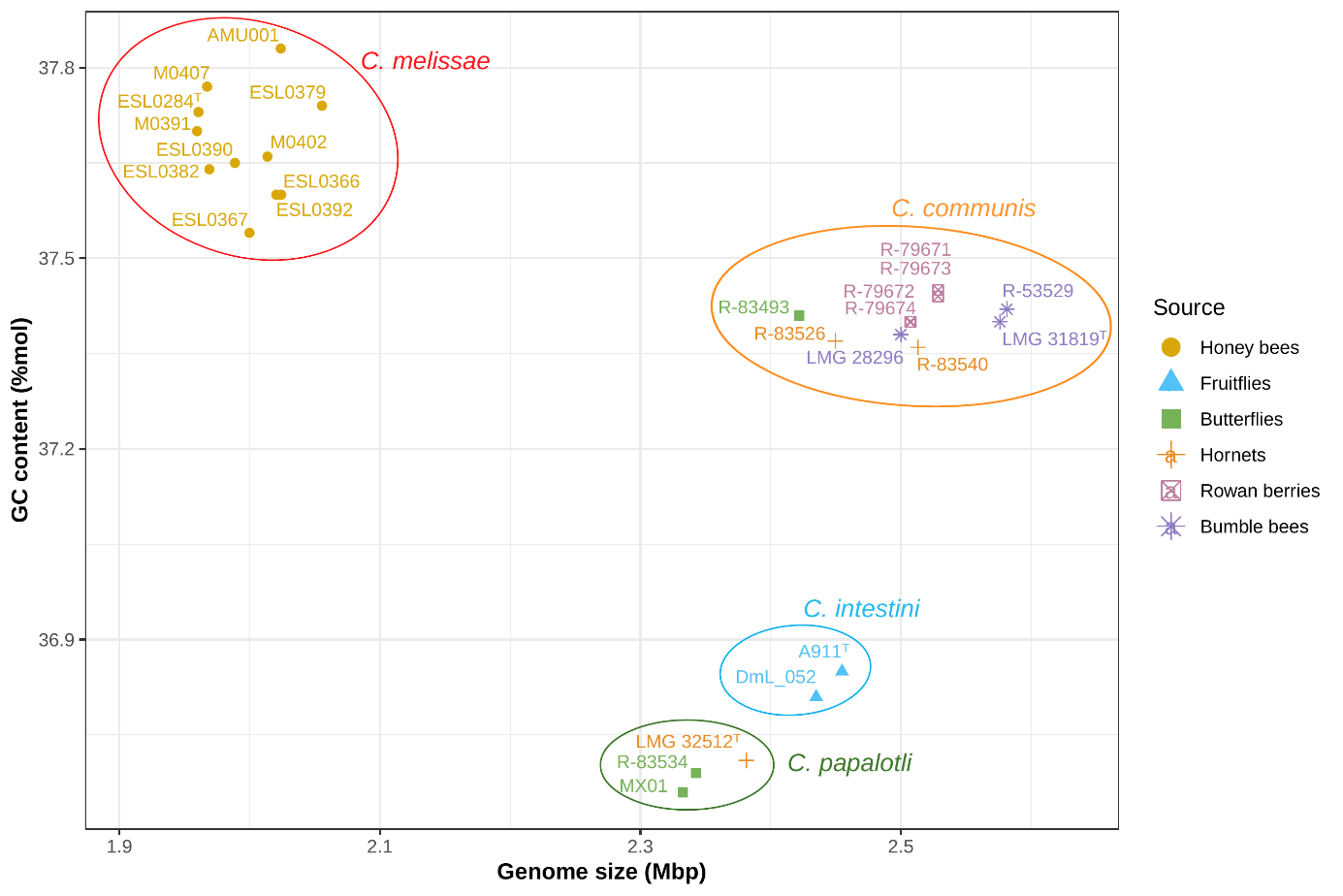


### Supplementary Figure S2. G + C content and genome size of *Commensalibacter* genomes. Isolates are color-coded by the isolation source. Circles indicate species clusters.


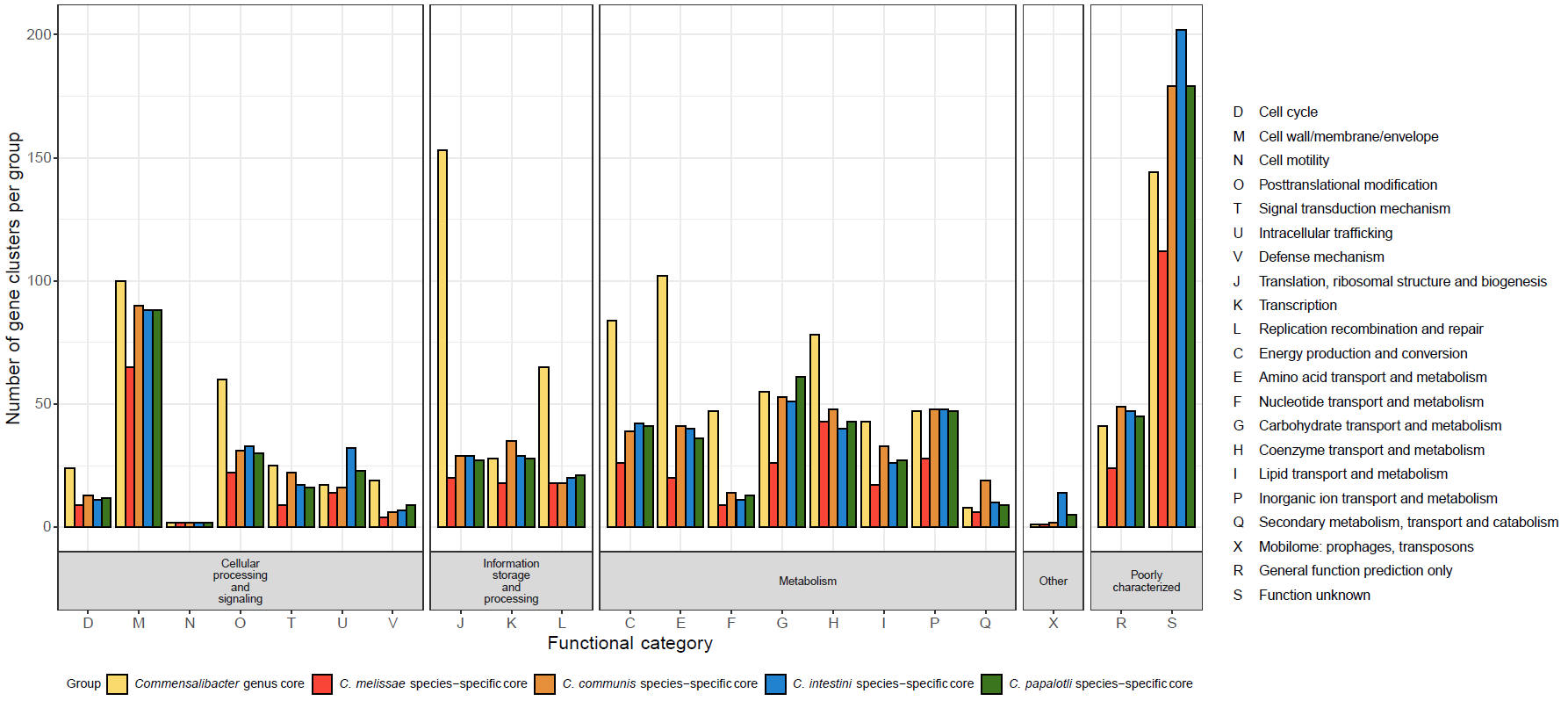


### Supplementary Figure S3. Distribution of gene clusters over COG categories of the *Commensalibacter* core and *Commensalibacter* species-specific core genomes. Numbers of gene clusters per group are presented.


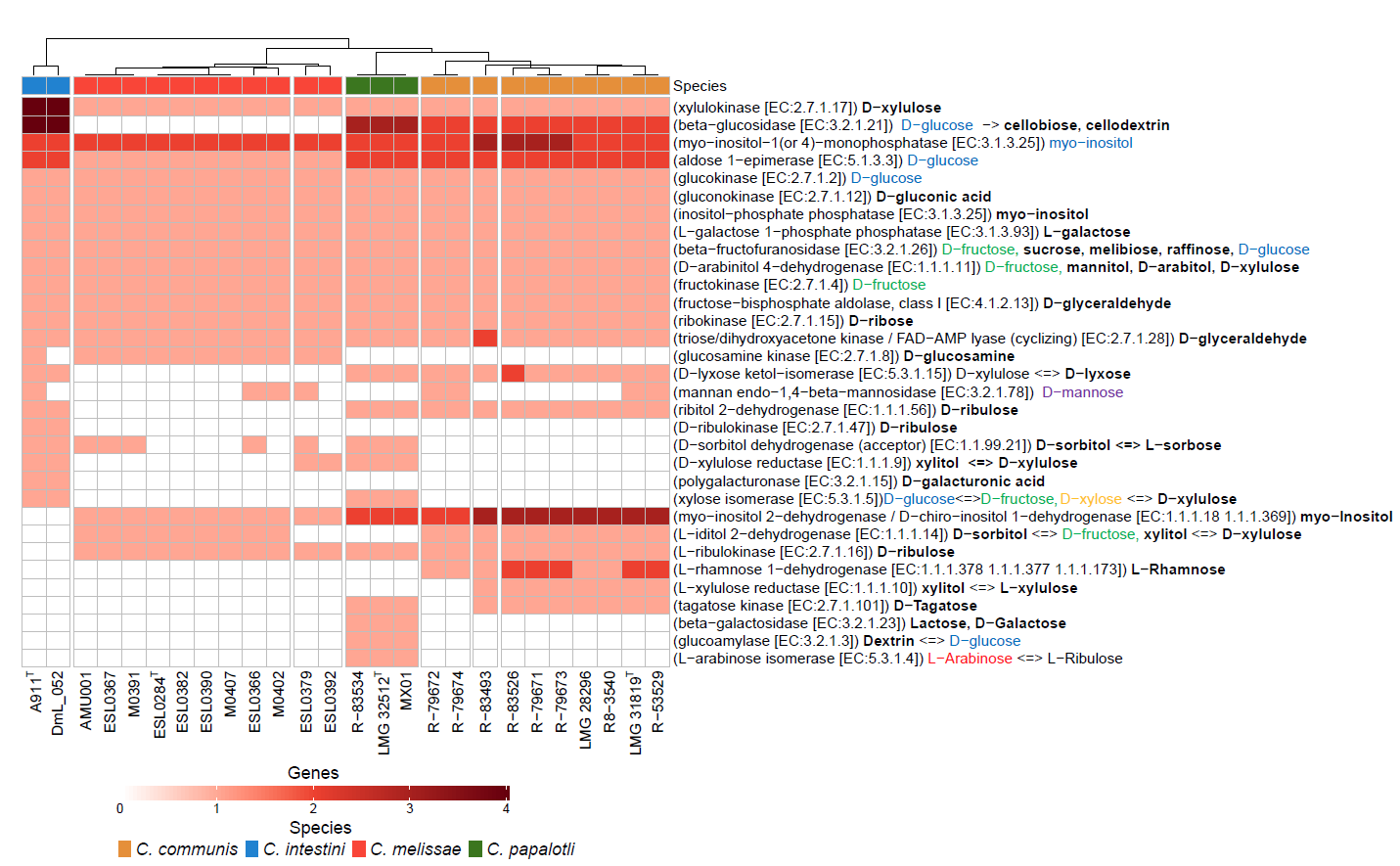


### Supplementary Figure S4. Heatmap showing the number of genes encoding carbohydrates utilizing enzymes based on the carbohydrate metabolism of the KEGG database. The scale ranges from zero to 4 indicating the number of genes or gene copies encoding carbohydrates utilizing enzymes. The dark and red colors represent the number of genes, while the white color represents the total absence of genes. The dendrogram shows that the isolates are clustered by species based on the Pearson correlation method.


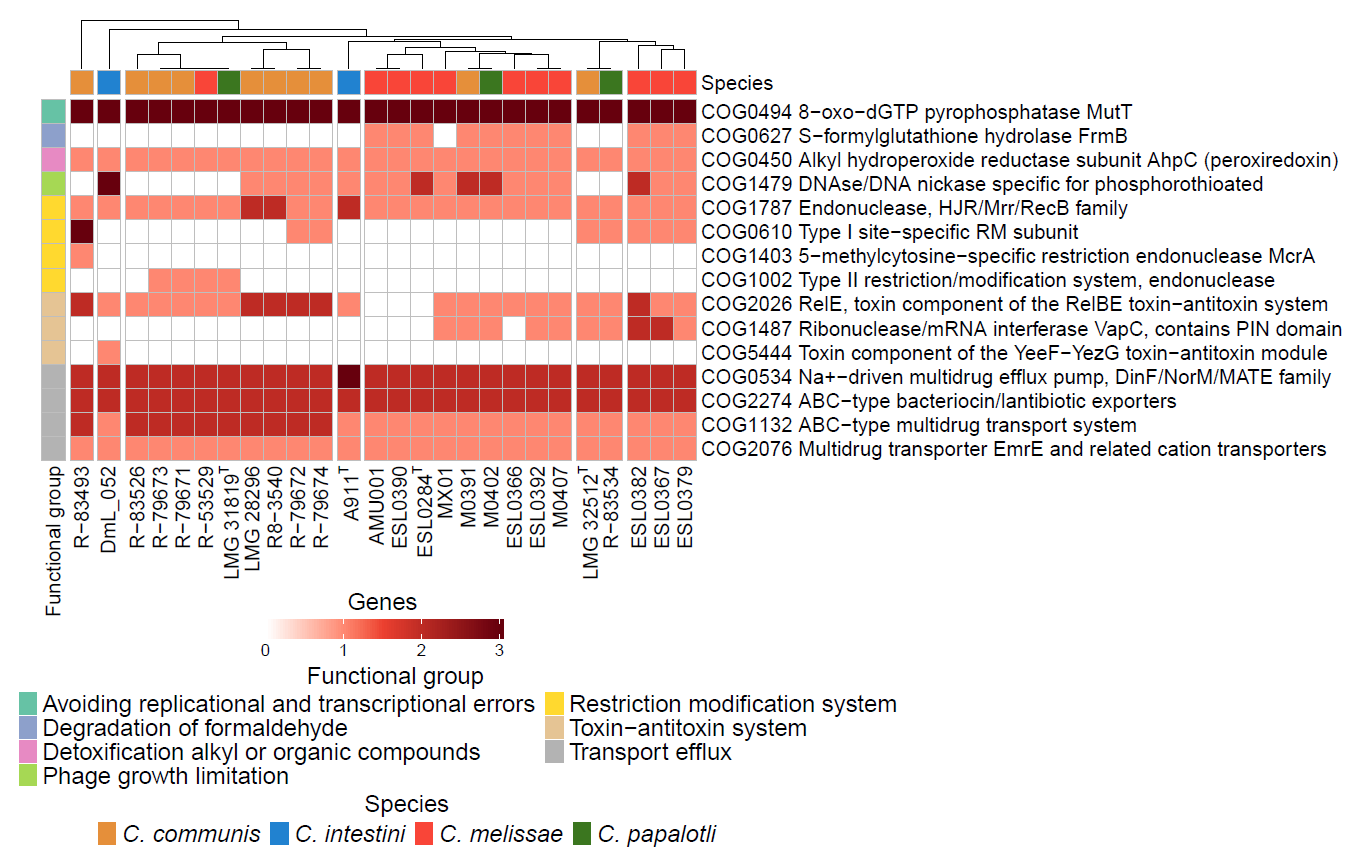


### Supplementary Figure S5. Heatmap displaying the number of genes belonging to the COG category Defense mechanism (V). The color scale ranges from zero (white) to 3 (dark red) indicating the number of genes in each COG number. The dendrogram shows that the isolates are clustered based on the Pearson correlation method.

Species descriptions

**Description of *Commensalibacter melissae* sp. nov.**

*Commensalibacter melissae* (me.lis’sae. Gr. fem. gen. n. *melissae*, from a bee).

Non-motile, gram-stain-negative short bacilli, about 1 µm wide and 2 µm long. Growth occurs on LMG agar medium 404 after 48h at 28°C, resulting in colonies that are flat, opaque, white and round, with an entire margin, and that are about 1 mm in diameter. Oxidase-negative. Catalase-positive. No ketogenesis from glycerol. Growth on SM with 1% NaCl and 2% NaCl. No growth on yeast extract with ammonia as the source of nitrogen and ethanol as the source of carbon. No growth in the presence of 30% d-glucose. No growth on 10% ethanol. No growth at pH 3.6. Growth on LMG agar medium 404 at 37°C. No production of 2-keto-d-gluconic acid, 5-keto-d-gluconic acid, and gluconic acid from d-glucose. No oxidation of ethanol.

No growth on TSA supplemented with 0.1% KNO_3_ under anaerobic conditions. Production of acid from d-arabinose, d-fructose, d-mannitol, d-mannose, d-ribose, dulcitol, ethanol, glycerol, melibiose, meso-erythritol, and d-sorbitol. Weak acid production from raffinose and sucrose. No production of acid from d-galactose, d-glucose or d-xylose. Growth on d-Ribose. No Growth on d-fructose, d-sorbitol, d-xylose, dulcitol, ethanol, glycerol, L-arabitol, maltose, meso-erythritol, methanol, raffinose, sodium acetate, and sucrose. Growth on d-sorbitol.

The type strain ESL0284^T^ (= LMG 31900^T^) was isolated in 2017 from a honey bee gut (*Apis mellifera*) sample. The 16S rRNA gene and whole-genome sequence of the strain ESL0284^T^ are publicly available through the accession numbers OX394067 and GCA_009734185, respectively. Its DNA G + C content is 37.73 mol%. Its genome size is 1.96 Mbp.

**Description of *Commensalibacter* *communis* sp. nov.**

*Commensalibacter communis* (com.mu’nis. L. fem. adj. *communis* common, because of its wide host range).

Non-motile, gram-stain-negative short bacilli, about 1 µm wide and 3 µm long. Growth occurs on LMG agar medium 404 after 48h at 28°C, resulting in colonies that are flat, opaque, white and round, with an entire margin, and that are about 1 mm in diameter. Oxidase-negative. Catalase-positive. No ketogenesis from glycerol. Growth on SM with 1% NaCl or 2% NaCl. No growth on yeast extract with ammonia as the source of nitrogen and ethanol as the source of carbon. Growth in the presence of 30% D-glucose. No growth on 10% ethanol. Growth at pH 3.6. Growth on LMG agar medium 404 at 37°C. No production of 2-keto-d-gluconic acid and 5-keto- d-gluconic acid from d-glucose. Production of gluconic acid from d-glucose. No oxidation of ethanol. Weak growth on TSA supplemented with 0.1% KNO_3_ under anaerobic conditions. Production of acid from d-glucose, d-mannitol, d-mannose, dulcitol, raffinose, sucrose and d-sorbitol. No production of acid from d-arabinose, galactose, d-fructose, d-ribose, d-xylose, ethanol, glycerol, melibiose, and meso-erythritol. Growth on d-sorbitol, d-xylose, dulcitol, glycerol, maltose, meso-erythritol, methanol, raffinose, sodium acetate, and sucrose. No growth on d-fructose, d-ribose, ethanol, and L-arabitol,

The type strain LMG 31819^T^ (= CECT 30152^T^) was isolated in 2013 from a *Bombus hypnorum* bumble bee gut sample. The 16S rRNA gene and whole-genome sequence of the strain LMG 31819^T^ are publicly available through the accession numbers OX394065 and GCA_947179015.1, respectively. Its DNA G + C content is 37.40 mol%. Its genome size is 2.57 Mbp.

**Description of *Commensalibacter* *papalotli* sp. nov.**

*Commensalibacter papalotli* (pa.pa.lot’li. N.L. gen. neut. n. *papalotli*, of papalotl which, in the mexican náhuatl language, means butterfly; named after the isolation source of the first reported isolate).

Non-motile, gram-stain-negative short bacilli, about 1 µm wide and 2 µm long. Growth occurs on LMG agar medium 404 after 48 h at 28°C, resulting in colonies that are flat, opaque, white and round, with an entire margin, with 1 mm in diameter. Oxidase-negative. Catalase-positive. No ketogenesis from glycerol. Growth on SM with 1% NaCl or 2% NaCl. No growth on yeast extract with ammonia as the source of nitrogen and ethanol as the source of carbon. Growth in the presence of 30% D-glucose. No growth on 10% ethanol. No growth at pH 3.6. No growth on LMG agar medium 404 at 37°C. No production of 2-keto-d-gluconic acid and 5-keto-d-gluconic acid from d-glucose. Production of gluconic acid from d-glucose. No oxidation of ethanol. Growth on TSA supplemented with 0.1% KNO_3_ and incubated under anaerobic conditions. Production of acid from d-arabinose, d-galactose, d-fructose, d-mannitol, d-mannose, d-ribose, dulcitol, ethanol, glycerol, melibiose, meso-erythritol, raffinose, sucrose, and d-sorbitol. Weak acid production from d-xylose. No production of acid from d-glucose. Growth on d-fructose, d-Ribose, d-sorbitol, d-xylose, dulcitol, ethanol, l-arabitol, maltose, meso-erythritol, methanol, raffinose, sodium acetate, and sucrose. No growth on glycerol.

The type strain LMG 32512^T^ (= CECT 30578^T^) was isolated from the midgut of a female hornet (*Vespa velutina*) in 2019. The 16S rRNA gene and whole-genome sequence of the strain LMG 32512^T^ are publicly available through the accession numbers OX394066 and GCA_947179115.1, respectively. Its DNA G + C content is 36.71 mol%. Its genome size is 2.38 Mbp.
