# Supplementary material for: A phylogenomic and comparative genomic analysis of Commensalibacter, a versatile insect symbiont
Source: Anim Microbiome. 2023 Apr 29;5:25. doi: 10.1186/s42523-023-00248-6 (PMC10149009; doi:10.1186/s42523-023-00248-6)
Supplement: Supplementary file 2 — Supplementary Material 2 [file 42523_2023_248_MOESM2_ESM.docx]

**Supplementary Table S4.** **Overview of formally named species corresponding with bee symbiont phylotypes.**

| **Taxon** | **Host** | **Reference** |  |
| --- | --- | --- | --- |
| **Core genera** |  |  | |
| ***Snodgrassella* (Beta)** |  |  | |
| *S. alvi* | Honey bee* | (Kwong and Moran 2013) | |
| *S. gandavensis* | Bumble bee | (Cornet, Cleenwerck et al. 2022) | |
| *S. communis* | Bumble bee | (Cornet, Cleenwerck et al. 2022) | |
|  |  |  | |
| ***Gilliamella* (Gamma-1)** |  |  | |
| *G. apicola* | Honey bee | (Kwong and Moran 2013) | |
| *G. apis* | Honey bee | (Ludvigsen, Porcellato et al. 2018) | |
| *G. bombi* | Bumble bee | (Praet, Cnockaert et al. 2017) | |
| *G. bombicola* | Bumble bee | (Praet, Cnockaert et al. 2017) | |
| *G. intestini* | Bumble bee | (Praet, Cnockaert et al. 2017) | |
| *G. mensalis* | Bumble bee | (Praet, Cnockaert et al. 2017) | |
|  |  |  | |
| ***Lactobacillus* (Firm-5** |  |  | |
| *L. apis* | Honey bee, bumble bee | (Killer, Dubna et al. 2014, Praet, Parmentier et al. 2018) | |
| *L. bombicola* | Bumble bee | (Praet, Meeus et al. 2015) | |
| *L. helsingborgensis* | Honey bee | (Olofsson, Alsterfjord et al. 2014) | |
| *L. huangpiensis* | Honey bee | (Li and Gu 2022) | |
| *L. kimbladii* | Honey bee | (Olofsson, Alsterfjord et al. 2014) | |
| *L. kullabergensis* | Honey bee | (Olofsson, Alsterfjord et al. 2014) | |
| *L. laiwuensis* | Honey bee | (Li and Gu 2022) | |
| *L. melliventris* | Honey bee | (Olofsson, Alsterfjord et al. 2014) | |
| *L. panisapium* | Honey bee (*A. cerana*) | (Wang, Huang et al. 2018) | |
|  |  |  | |
| ***Bombilactobacillus* (Firm-4)** |  |  | |
| *B. apium* | Honey bee (*A. cerana*) | (Kang, Huo et al. 2021) | |
| *B.* *bombi* | Bumble bee | (Killer, Votavova et al. 2014) | |
| *B.* *folatiphilus* | Stingless bee | (Oliphant, Watson-Haigh et al. 2022) | |
| *B.* *mellifer* | Honey bee | (Olofsson, Alsterfjord et al. 2014) | |
| *B.* *mellis* | Honey bee | (Olofsson, Alsterfjord et al. 2014) | |
| *B.* *thymidiniphilus* | Stingless bee | (Oliphant, Watson-Haigh et al. 2022) | |

| ***Bifidobacterium* (Bifido)** |  |  |
| --- | --- | --- |
| *B. actinocoloniiforme* | Bumble bee | (Killer, Kopecny et al. 2011) |
| *B. aemilianum* | Carpenter bee | (Alberoni, Gaggia et al. 2019) |
| *B. apousia* | Honey bee | (Chen, Wang et al. 2021) |
| *B.* *asteroides* | Honey bee | (Milani, Lugli et al. 2014) |
| *B.* *bohemicum* | Bumble bee | (Killer, Kopecny et al. 2011) |
| *B.* *bombi* | Bumble bee | (Killer, Kopecny et al. 2009) |
| *B. choladohabitans* | Honey bee | (Chen, Wang et al. 2021) |
| *B.* *commune* | Bumble bee | (Praet, Meeus et al. 2015) |
| *B.* *coryneforme* | Honey bee | (Milani, Lugli et al. 2014) |
| *B.* *indicum* | Honey bee | (Milani, Lugli et al. 2014) |
| *B.* *mellis* | Honey bee | (Olofsson, Modesto et al. 2023) |
| *B.* *mizhiense* | Honey bee | (Li, Zhang et al. 2022) |
| *B. polysaccharolyticum* | Honey bee | (Chen, Wang et al. 2021) |
| *B. xylocopae* | Carpenter bee | (Alberoni, Gaggia et al. 2019) |
|  |  |  |
| **Non-core genera** |  |  |
| ***Apibacter* (Bacteroides)** |  |  |
| *A. adventoris* | Honey bee | (Kwong and Moran 2016) |
| *A. mensalis* | Bumble bee | (Praet, Aerts et al. 2016) |
|  |  |  |
| ***Bartonella* (Alpha-1)** |  |  |
| *B. apis* | Honey bee | (Kekerova, Moritz et al. 2016) |
| *B. apihabitans* | Honey bee | (Liu, Chen et al. 2022) |
| *B. choladocola* | Honey bee | (Liu, Chen et al. 2022) |
|  |  |  |
| ***Commensalibacter* (Alpha-2.1)** |  |  |
| *C. communis* | Bumble bee, butterfly, Asian hornet, environmental | The present study |
| *C. intestini* | Fruit fly | (Roh, Nam et al. 2008) |
| *C. melissae* | Honey bee | The present study |
| *C. papalotli* | Butterfly, Asian hornet | The present study |
|  |  |  |
| ***Bombella* (Alpha-2.2)** |  |  |
| *B. apis* | Honey bee | (Yun, Lee et al. 2017) |
| *B. favorum* | Honey bee | (Hilgarth, Redwitz et al. 2021) |
| *B. mellum* | Honey bee | (Hilgarth, Redwitz et al. 2021) |
| *B. intestini* | Bumble bee | (Li, Praet et al. 2015) |
|  |  |  |
| ***Bombiscardovia*** **(Bifido)** |  |  |
| *B. coagulans* | Bumble bee | (Killer, Kopecny et al. 2010) |
|  |  |  |
| ***Frischella* (Gamma-2)** |  |  |
| *F. perrara* | Honey bee | (Engel, Kwong et al. 2013) |
| *F. japonica* | Honey bee (*A. cerana*) | (Wolter, Suenami et al. 2021) |
|  |  |  |
| ***Apilactobacillus* (Lacto-3)** |  |  |
| *A. apinorum* | Honey bee | (Olofsson, Alsterfjord et al. 2014) |
| *A. apisilvae* | Stingless bee | (Oliphant, Watson-Haigh et al. 2022) |
| *A. bombintestini* | Bumble bee | (Heo, Kim et al. 2020) |
| *A. micheneri* | Sweat bee | (McFrederick, Vuong et al. 2018) |
| *A. kunkeei* | Honey bee, bumble bee | (Anderson, Rodrigues et al. 2016, Martijn, Lind et al. 2019) |
| *A. nanyangensis* | Honey bee | (Liu, Li et al. 2021) |
| *A. queniae* | Sweat bee | (McFrederick, Vuong et al. 2018) |
| *A. timberlakei* | Sweat bee | (McFrederick, Vuong et al. 2018) |
| *A. xinyiensis* | Honey bee | (Li and Gu 2022) |
| *A. zhangqiuensis* | Honey bee | (Li and Gu 2022) |
| **Apis mellifera* if not specified. |  |  |
|  |  |  |
|  |  |  |
|  |  |  |

**References cited**

Alberoni, D., F. Gaggia, L. Baffoni, M. M. Modesto, B. Biavati and D. Di Gioia (2019). "Bifidobacterium xylocopae sp. nov. and Bifidobacterium aemilianum sp. nov., from the carpenter bee (Xylocopa violacea) digestive tract." Systematic and Applied Microbiology **42**(2): 205-216.

Anderson, K. E., P. A. P. Rodrigues, B. M. Mott, P. Maes and V. Corby-Harris (2016). "Ecological Succession in the Honey Bee Gut: Shift in Lactobacillus Strain Dominance During Early Adult Development." Microbial Ecology **71**(4): 1008-1019.

Chen, J. T., J. N. Wang and H. Zheng (2021). "Characterization of Bifidobacterium apousia sp. nov., Bifidobacterium choladohabitans sp. nov., and Bifidobacterium polysaccharolyticum sp. nov., three novel species of the genus Bifidobacterium from honey bee gut." Systematic and Applied Microbiology **44**(5).

Cornet, L., I. Cleenwerck, J. Praet, R. R. Leonard, N. J. Vereecken, D. Michez, G. Smagghe, D. Baurain and P. Vandamme (2022). "Phylogenomic Analyses of Snodgrassella Isolates from Honeybees and Bumblebees Reveal Taxonomic and Functional Diversity." Msystems **7**(3).

Engel, P., W. K. Kwong and N. A. Moran (2013). "Frischella perrara gen. nov., sp nov., a gammaproteobacterium isolated from the gut of the honeybee, Apis mellifera." International Journal of Systematic and Evolutionary Microbiology **63**: 3646-3651.

Heo, J., S. J. Kim, J. S. Kim, S. B. Hong and S. W. Kwon (2020). "Comparative genomics of Lactobacillus species as bee symbionts and description of Lactobacillus bombintestini sp. nov., isolated from the gut of Bombus ignitus." Journal of Microbiology **58**(6): 445-455.

Hilgarth, M., J. Redwitz, M. A. Ehrmann, R. F. Vogel and F. Jakob (2021). "Bombella favorum sp. nov. and Bombella mellum sp. nov., two novel species isolated from the honeycombs of Apis mellifera." International Journal of Systematic and Evolutionary Microbiology **71**(2).

Kang, J. P., Y. Huo, V. A. Hoang, D. U. Yang, D. C. Yang and S. C. Kang (2021). "Bombilactobacillus apium sp. nov., isolated from the gut of honeybee (Apis cerana)." Archives of microbiology **203**(5): 2193-2198.

Kekerova, L., R. Moritz and P. Engel (2016). "Bartonella apis sp nov., a honey bee gut symbiont of the class Alphaproteobacteria." International Journal of Systematic and Evolutionary Microbiology **66**: 414-421.

Killer, J., S. Dubna, I. Sedlacek and P. Svec (2014). "Lactobacillus apis sp. nov., from the stomach of honeybees (Apis mellifera), having an in vitro inhibitory effect on the causative agents of American and European foulbrood." International journal of systematic and evolutionary microbiology **64**(Pt 1): 152-157.

Killer, J., J. Kopecny, J. Mrazek, J. Havlik, I. Koppova, O. Benada, V. Rada and O. Kofronova (2010). "Bombiscardovia coagulans gen. nov., sp. nov., a new member of the family Bifidobacteriaceae isolated from the digestive tract of bumblebees." Systematic and applied microbiology **33**(7): 359-366.

Killer, J., J. Kopecny, J. Mrazek, I. Koppova, J. Havlik, O. Benada and T. Kott (2011). "Bifidobacterium actinocoloniiforme sp nov and Bifidobacterium bohemicum sp nov., from the bumblebee digestive tract." International Journal of Systematic and Evolutionary Microbiology **61**: 1315-1321.

Killer, J., J. Kopecny, J. Mrazek, V. Rada, O. Benada, I. Koppova, J. Havlik and J. Straka (2009). "Bifidobacterium bombi sp nov., from the bumblebee digestive tract." International Journal of Systematic and Evolutionary Microbiology **59**: 2020-2024.

Killer, J., A. Votavova, I. Valterova, E. Vlkova, V. Rada and Z. Hroncova (2014). "Lactobacillus bombi sp nov., from the digestive tract of laboratory-reared bumblebee queens (Bomb us terrestris)." International Journal of Systematic and Evolutionary Microbiology **64**: 2611-2617.

Kwong, W. K. and N. A. Moran (2013). "Cultivation and characterization of the gut symbionts of honey bees and bumble bees: description of Snodgrassella alvi gen. nov., sp nov., a member of the family Neisseriaceae of the Betaproteobacteria, and Gilliamella apicola gen. nov., sp nov., a member of Orbaceae fam. nov., Orbales ord. nov., a sister taxon to the order 'Enterobacteriales' of the Gammaproteobacteria." International Journal of Systematic and Evolutionary Microbiology **63**: 2008-2018.

Kwong, W. K. and N. A. Moran (2016). "Apibacter adventoris gen. nov., sp nov., a member of the phylum Bacteroidetes isolated from honey bees." International Journal of Systematic and Evolutionary Microbiology **66**: 1323-1329.

Li, L., J. Praet, W. Borremans, O. C. Nunes, C. M. Manaia, I. Cleenwerck, I. Meeus, G. Smagghe, L. De Vuyst and P. Vandamme (2015). "Bombella intestini gen. nov., sp. nov., an acetic acid bacterium isolated from bumble bee crop." International journal of systematic and evolutionary microbiology **65**(Pt 1): 267-273.

Li, T. T. and C. T. Gu (2022). "Apilactobacillus zhangqiuensis sp. nov. and Apilactobacillus xinyiensis sp. nov., isolated from the gut of honeybee (Apis mellifera)." International Journal of Systematic and Evolutionary Microbiology **72**(5).

Li, T. T. and C. T. Gu (2022). "Lactobacillus huangpiensis sp. nov. and Lactobacillus laiwuensis sp. nov., isolated from the gut of honeybee (Apis mellifera)." International Journal of Systematic and Evolutionary Microbiology **72**(2).

Li, T. T., H. X. Zhang and C. T. Gu (2022). "Bifidobacterium mizhiense sp. nov., isolated from the gut of honeybee (Apis mellifera)." International Journal of Systematic and Evolutionary Microbiology **72**(5).

Liu, D. D., Y. Q. Li, L. P. Zhang, W. Ding, W. L. Tian and C. T. Gu (2021). "Apilactobacillus nanyangensis sp. nov., Secundilactobacillus hailunensis sp. nov., Secundilactobacillus yichangensis sp. nov., Levilactobacillus andaensis sp. nov., Levilactobacillus wangkuiensis sp. nov., Levilactobacillus lanxiensis sp. nov., Lacticaseibacillus mingshuiensis sp. nov. and Lacticaseibacillus suilingensis sp. nov., isolated from traditional Chinese pickle and the gut of honeybee (Apis mellifera)." International Journal of Systematic and Evolutionary Microbiology **71**(7).

Liu, Y. W., J. T. Chen, H. Y. Lang and H. Zheng (2022). "Bartonella choladocola sp. nov. and Bartonella apihabitans sp. nov., two novel species isolated from honey bee gut." Systematic and Applied Microbiology **45**(6).

Ludvigsen, J., D. Porcellato, G. V. Amdam and K. Rudi (2018). "Addressing the diversity of the honeybee gut symbiont Gilliamella: description of Gilliamella apis sp nov., isolated from the gut of honeybees (Apis mellifera)." International Journal of Systematic and Evolutionary Microbiology **68**(5): 1762-1770.

Martijn, J., A. E. Lind, M. E. Schon, I. Spiertz, L. Juzokaite, I. Bunikis, O. V. Pettersson and T. J. G. Ettema (2019). "Confident phylogenetic identification of uncultured prokaryotes through long read amplicon sequencing of the 16S-ITS-23S rRNA operon." Environmental microbiology **21**(7): 2485-2498.

McFrederick, Q. S., H. Q. Vuong and J. A. Rothman (2018). "Lactobacillus micheneri sp nov., Lactobacillus timberlakei sp nov and Lactobacillus quenuiae sp nov., lactic acid bacteria isolated from wild bees and flowers." International Journal of Systematic and Evolutionary Microbiology **68**(6): 1879-1884.

Milani, C., G. A. Lugli, S. Duranti, F. Turroni, F. Bottacini, M. Mangifesta, B. Sanchez, A. Viappiani, L. Mancabelli, B. Taminiau, V. Delcenserie, R. Barrangou, A. Margolles, D. van Sinderen and M. Ventura (2014). "Genomic Encyclopedia of Type Strains of the Genus Bifidobacterium." Applied and Environmental Microbiology **80**(20): 6290-6302.

Oliphant, S. A., N. S. Watson-Haigh, K. M. Sumby, J. Gardner, S. Groom and V. Jiranek (2022). "Apilactobacillus apisilvae sp. nov., Nicolia spurrieriana gen. nov. sp. nov., Bombilactobacillus folatiphilus sp. nov. and Bombilactobacillus thymidiniphilus sp. nov., four new lactic acid bacterial isolates from stingless bees Tetragonula carbonaria and Austroplebeia austratis." International Journal of Systematic and Evolutionary Microbiology **72**(9).

Olofsson, T. C., M. Alsterfjord, B. Nilson, E. Butler and A. Vasquez (2014). "Lactobacillus apinorum sp nov., Lactobacillus mellifer sp nov., Lactobacillus mellis sp nov., Lactobacillus melliventris sp nov., Lactobacillus kimbladii sp nov., Lactobacillus helsingborgensis sp nov and Lactobacillus kullabergensis sp nov., isolated from the honey stomach of the honeybee Apis mellifera." International Journal of Systematic and Evolutionary Microbiology **64**: 3109-3119.

Olofsson, T. C., M. Modesto, S. Pascarelli , D. Scarafile , P. Mattarelli and A. Vasquez (2023). "Bifidobacterium mellis sp. nov., isolated from the honey stomach of the honey bee Apis mellifera. ." nt J Syst Evol Microbiol **73**: 5766.

Praet, J., M. Aerts, E. De Brandt, I. Meeus, G. Smagghe and P. Vandamme (2016). "Apibacter mensalis sp nov.: a rare member of the bumblebee gut microbiota." International Journal of Systematic and Evolutionary Microbiology **66**: 1645-1651.

Praet, J., M. Cnockaert, I. Meeus, G. Smagghe and P. Vandamme (2017). "Gilliamella intestini sp nov., Gilliamella bombicola sp nov., Gilliamella bombi sp nov and Gilliamella mensalis sp nov.: Four novel Gilliamella species isolated from the bumblebee gut." Systematic and Applied Microbiology **40**(4): 199-204.

Praet, J., I. Meeus, M. Cnockaert, M. Aerts, G. Smagghe and P. Vandamme (2015). "Bifidobacterium commune sp nov isolated from the bumble bee gut." Antonie Van Leeuwenhoek International Journal of General and Molecular Microbiology **107**(5): 1307-1313.

Praet, J., I. Meeus, M. Cnockaert, K. Houf, G. Smagghe and P. Vandamme (2015). "Novel lactic acid bacteria isolated from the bumble bee gut: Convivina intestini gen. nov., sp nov., Lactobacillus bombicola sp nov., and Weissella bombi sp nov." Antonie Van Leeuwenhoek International Journal of General and Molecular Microbiology **107**(5): 1337-1349.

Praet, J., A. Parmentier, R. Schmid-Hempel, I. Meeus, G. Smagghe and P. Vandamme (2018). "Large-scale cultivation of the bumblebee gut microbiota reveals an underestimated bacterial species diversity capable of pathogen inhibition." Environmental microbiology **20**(1): 214-227.

Roh, S. W., Y. D. Nam, H. W. Chang, K. H. Kim, M. S. Kim, J. H. Ryu, S. H. Kim, W. J. Lee and J. W. Bae (2008). "Phylogenetic characterization of two novel commensal bacteria involved with innate immune homeostasis in Drosophila melanogaster." Applied and Environmental Microbiology **74**(20): 6171-6177.

Wang, C., Y. Huang, L. Li, J. Guo, Z. Y. Wu, Y. Deng, L. R. Dai and S. C. Ma (2018). "Lactobacillus panisapium sp nov., from honeybee Apis cerana bee bread." International Journal of Systematic and Evolutionary Microbiology **68**(3): 703-708.

Wolter, L. A., S. Suenami and R. Miyazaki (2021). "Frischella japonica sp. nov., an anaerobic member of the Orbales in the Gammaproteobacteria, isolated from the gut of the eastern honey bee, Apis cerana japonica Fabricius." International Journal of Systematic and Evolutionary Microbiology **71**(3).

Yun, J. H., J. Y. Lee, D. W. Hyun, M. J. Jung and J. W. Bae (2017). "Bombella apis sp nov., an acetic acid bacterium isolated from the midgut of a honey bee." International Journal of Systematic and Evolutionary Microbiology **67**(7): 2184-2188.
